# Evaluation of fNIRS signal components elicited by cognitive and hypercapnic stimuli

**DOI:** 10.1038/s41598-021-02076-7

**Published:** 2021-12-06

**Authors:** Pratusha Reddy, Meltem Izzetoglu, Patricia A. Shewokis, Michael Sangobowale, Ramon Diaz-Arrastia, Kurtulus Izzetoglu

**Affiliations:** 1 School of Biomedical Engineering, Science and Health Systems, Drexel University, Philadelphia, PA 19104 USA; 2 Department of Electrical and Computer Engineering, Villanova University, Villanova, PA 19085 USA; 3 Nutrition Sciences Department of College of Nursing and Health Professions, Drexel University, Philadelphia, PA 19104 USA; 4 Department of Teaching, Learning and Curriculum, School of Education, Drexel University, Philadelphia, PA 19104 USA; 5 Clinical TBI Research Center and Department of Neurology, University of Pennsylvania Perelman School of Medicine, Philadelphia, PA 19104 USA

**Keywords:** Cognitive neuroscience, Neuro-vascular interactions, Neuronal physiology

## Abstract

Functional near infrared spectroscopy (fNIRS) measurements are confounded by signal components originating from multiple physiological causes, whose activities may vary temporally and spatially (across tissue layers, and regions of the cortex). Furthermore, the stimuli can induce evoked effects, which may lead to over or underestimation of the actual effect of interest. Here, we conducted a temporal, spectral, and spatial analysis of fNIRS signals collected during cognitive and hypercapnic stimuli to characterize effects of functional versus systemic responses. We utilized wavelet analysis to discriminate physiological causes and employed long and short source-detector separation (SDS) channels to differentiate tissue layers. Multi-channel measures were analyzed further to distinguish hemispheric differences. The results highlight cardiac, respiratory, myogenic, and very low frequency (VLF) activities within fNIRS signals. Regardless of stimuli, activity within the VLF band had the largest contribution to the overall signal. The systemic activities dominated the measurements from the short SDS channels during cognitive stimulus, but not hypercapnic stimulus. Importantly, results indicate that characteristics of fNIRS signals vary with type of the stimuli administered as cognitive stimulus elicited variable responses between hemispheres in VLF band and task-evoked temporal effect in VLF, myogenic and respiratory bands, while hypercapnic stimulus induced a global response across both hemispheres.

## Introduction

Over the past two decades, functional near infrared spectroscopy (fNIRS) has emerged as an effective and viable modality to study brain activity during various cognitive and motor tasks or at resting states in healthy and diseased populations with acquired pathologies (i.e. Traumatic Brain Injury, Stroke) or induced stimuli (i.e. Carbon Dioxide – CO_2_, sevoflurane)^1–6^. Increases in metabolic demand within the brain is coupled with an increase in oxygen consumption and cerebral blood flow (CBF), which leads to highly correlated changes in oxygenated hemoglobin (HbO) and deoxygenated hemoglobin (HbR) that is readily detectable by fNIRS^7,8^. Although many studies have validated the sensitivity of fNIRS in relation to functional magnetic resonance imaging (fMRI) results, some studies have noted false positives and false negatives in fNIRS activation maps^9,10^. These results have been attributed to variation in head anatomy, physiology, and functioning and the near infrared (NIR) light propagation within this complex structure.

In cognitive studies, task-related vasodilation stemming from neuronal origins within the cerebral cortex as a result of neurovascular coupling (NVC) is the signal of interest^11^. Alternatively, in cerebral autoregulation (CA) or cerebrovascular reactivity (CVR) studies, changes originating from systemic factors, such as blood pressure or partial arterial CO_2_ (PaCO_2_) are the signals of interest^12,13^. However, regardless of stimuli, fNIRS measurements are composed of signal components originating from different: (i) physiological causes (i.e. neuronal vs systemic), (ii) spatial locations (axially for tissue layers i.e. cerebral vs extracerebral or laterally i.e. left vs right hemispheres), and (iii) experimental or task related elements (i.e. evoked vs resting state or with vs. without stressors)^14–18^. Therefore, extraction of the signal of interest requires application of appropriate signal processing techniques, which first entails thorough characterization and analysis of signal components arising because of the task being studied.

Systemic factors associated with cardiac, respiration, and myogenic (oscillations of the sympathetic vasomotor tone and blood pressure regulation mechanism by autonomic nervous system-ANS and baroreceptors) contribute at least 35% to fNIRS signals^19^. Cardiac, respiratory, and myogenic activities have been shown to appear within unique frequency bands of 0.6 to 1.6 Hz, 0.2 to 0.4 Hz, and 0.06 to 0.1 Hz, respectively^20,21^. Systemic activity associated with PaCO_2_ shares the same band of 0.01 to 0.06 Hz as functional/neuronal activity, and has been projected to contribute at least 1.5% to fNIRS signals^22–24^. The simplest and the most utilized class of methods to remove frequencies associated with cardiac, respiratory and myogenic activities, consist of applying a band pass (cut-off frequencies at 0.01 and 0.09 Hz) or low pass (cut-off frequency at 0.09 Hz) Finite Impulse Response, or Butterworth filters^25^. Other filters such as Gaussian, Kalman, Wiener and hemodynamic response filters have also been commonly used to remove aforementioned systemic noises in real time or off-line signal processing applications^26^. However, such filters could suppress some of the task-related neuronal effects due to non-ideal filter characteristics and assumptions used in filter designs. Methods utilizing additional systemic physiological sensors such as electrocardiography, laser doppler flowmetry and others can be used for similar purposes^27–30^. However, addition of such sensors requires time synchronization between different sensors and may be cumbersome on the participant. Furthermore, these methods are unable to remove PaCO_2_, or extracerebral contributions^31,32^.

Due to the physics of photon migration within the head, sensitivity of NIR light is affected by various extracerebral (skin, scalp, skull, and cerebrospinal fluid) and cerebral layers (gray and white matter) of different optical characteristics and thickness. A comprehensive examination on the percentage of light absorbed by different brain layers in healthy and clinical conditions revealed that at best, only ~ 18% of light was absorbed by cerebral layers^33^. Without additional hardware requirements, blind source separation techniques such as Independent Component Analysis (ICA) or Principle Component Analysis (PCA) can be used to suppress extracerebral signals using multi-channel fNIRS measurements^34–36^. These techniques require assumptions regarding uncorrelatedness (as in PCA), orthogonality and statistical independence (as in ICA) between components to be guaranteed. However, such assumptions are typically violated when skin perfusion changes are also modulated by the task^28^. Alternatively, methods utilizing the addition of short source-detector separation (SDS) channels (0.5–1.0 cm separation) to the fNIRS sensor alongside the long SDS channels (2–4 cm separation) allow for direct separation of signals that have penetrated the cerebral layer and those that have not. The most direct techniques that utilize short SDS channels are the least squares adaptive filters and regression modelling^37–41^. Although these techniques improve contrast-to-noise ratio by 70%, they assume that extracerebral contributions are static and spatially homogeneous, which is not always accurate^28,35,42^. Independent of task, characteristics of extracerebral signals may vary spatially across different regions of the head due to the differences in terms of geometry and composition of tissue layers^34,43^. Kalman filter and state-space model-based methods help account for the non-stationary nature of hemodynamics^44,45^. Addition of multiple short SDS channels placed at varying length from a single detector allow for accountancy of spatial heterogeneity^42,46^.

The magnitude and frequencies of all the aforementioned fNIRS signal components may be influenced by the specific experimental tasks being used to investigate cognition, psychosocial stress, emotional state, or induced physiological stressors^28,29,47–49^. Furthermore, it is proposed that neuronal and systemic factors arising from cerebral and extracerebral layers do not take place as isolated processes but come from an interactive network of interlinked processes^50,51^. For example, studies have demonstrated significant changes in myogenic activity during motor and cognitive tasks in cerebral and extracerebral layers^52,53^. Others have shown that PaCO_2_ induced CBF changes can lead to an underestimation of NVC resulting CBF changes during out loud and inner speech-oriented cognitive tasks^54^. In a cognitively challenging n-back study, not removing extracerebral contribution from fNIRS measurements led to insignificant changes in functional activation with task difficulty^55^. However, removal of extracerebral contribution led to a decrease in HbO measures due to increases in skin conductance and heart rate, and an increase in HbO in the cerebral compartment due to NVC. While in some experimental protocols it is imperative to remove these systemic or extracerebral effects to obtain task-evoked cerebral neuronal signals, in other cases they can be treated as additional signals of interest as they may carry complementary information regarding the state of the subject^56,57^. For example, one study added time and frequency domain features of cardiac oscillations extracted from fNIRS signals to a machine learning architecture and reported increases in classification accuracy of stress by at least 10% when compared against standard models containing only the neuronal activity related information^58^. Another study reported that including end-tidal CO_2_ (EtCO_2_—surrogate for PaCO_2_ measures) in the interpretation of fNIRS signals improved ability to monitor pain^59^. These findings indicate that to comprehensively evaluate cerebral hemodynamic changes due to a task/stimulus, it is important to not just remove the non-neuronal effects, but to analyze contribution of each of the signal components to the fNIRS measurements, separately and in relation to each other.

Time frequency analysis serves as a good analytical tool to investigate fNIRS signal components within the same plane. The wavelet transform (WT) provides windows of adjustable length thereby ensuring good resolution for both high and low frequency content^60^. WT has been previously used in fNIRS signal processing to remove motion artifacts, and global drifts^61,62^. In fact, comparisons of the wavelet filter against the band pass filter, indicated the wavelet filter improved temporal consistency and spatial specificity over conventional methods^63^. WT analysis of fNIRS signals collected from prefrontal cortex (PFC) of healthy individuals have reported presence of local maxima in cardiac, respiratory, myogenic, and very low frequency (VLF – PaCO_2_ or neuronal related activity) bands^29,64–66^. Alterations in amplitude and frequency of these factors as indicated by WT analysis have been reported in studies investigating healthy aging, stroke, and cerebral infraction^23,67–69^. However, existing studies have not used WT analysis on measurements taken from cerebral and extracerebral layers or applied the technique to compare evoked versus non-evoked activity or evaluated differences across regions of the PFC.

The main aim in this work was to quantify the contribution of signal components from different origins including physiological causes, tissue layers and hemispheres on fNIRS recordings collected under different experimental paradigms. To achieve this goal, we used WT, long and short SDS channels, and multi-channel measurements. The data used here was acquired from two different studies. One study gathered data from a complex cognitive task, while the other one collected data while inducing hypercapnia^70,71^. The cognitive study employed realistic search and surveillance task scenarios on novice unmanned aircraft system (UAS) sensor operators. This experiment was chosen because preliminary results have shown strong NVC activity in left and right PFC regions associated with working memory and sustained attention^72^. In the second study, hypercapnia was the chosen stimulus, since it is known to result in a homogenous effect across all regions of PFC, does not require subject engagement and does not induce evoked NVC^71^. More importantly, the hypercapnic task allows for quantification of PaCO_2_’s effect.

## Methods

### Study I: cognitive stimuli

**Figure 1:**
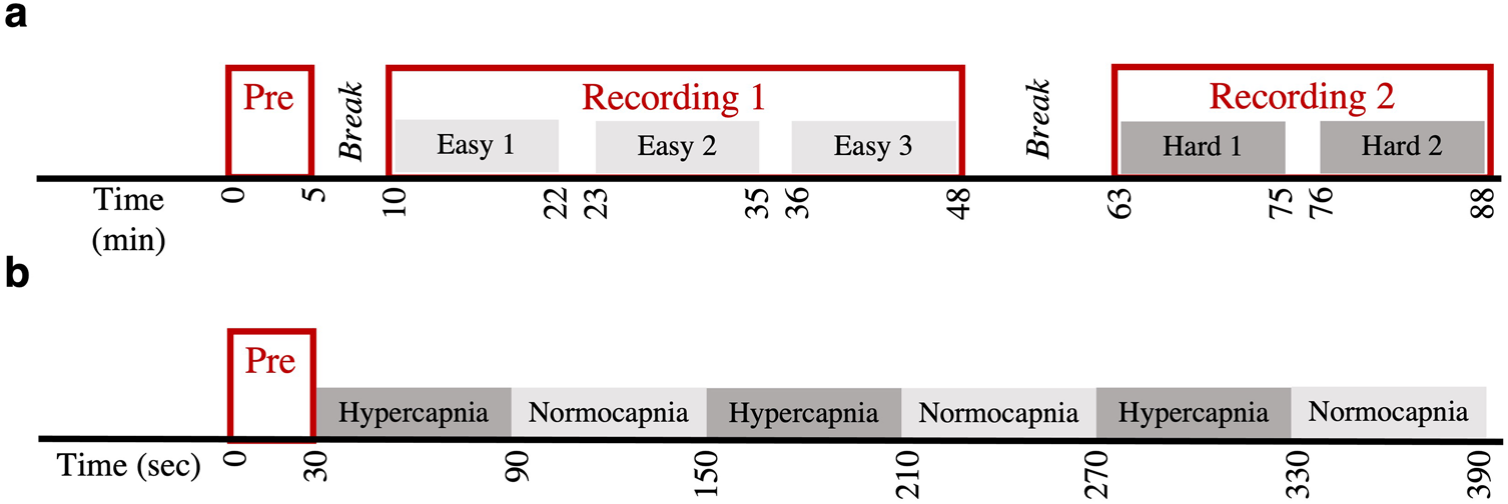
Experimental protocols. (**a**) In cognitive task, each participant started with a brief instructional session regarding UAS controls and task objectives, followed by five 12 min task conditions of varying difficulties. The first three task conditions were of easier difficulty, while the last two were of harder difficulty. A 5 and 15-min breaks were given between instructional and first easy condition, and last easy and first hard condition. (**b**) In hypercapnic task, each participant began with a 30 s baseline period (Room Air – 0.04% CO_2_), followed by three hypercapnic (5% CO_2_) and normocapnic (Room Air) conditions of 60 s each.

Thirteen participants between the ages of 19 and 40 (3 female) were recruited to take part in this study. All participants provided written informed consent approved by the Institutional Review Board (IRB) of Drexel University. The research was performed in accordance with relevant regulations. Inclusion criteria required no prior UAS piloting simulator experience, normal or corrected to normal vision, and right handedness verified via the Edinburgh Handedness assessment^73^. A high-fidelity ground station simulator, the Simlat’s C-STAR (Simlat Inc., Miamisburg, Ohio), was used to implement realistic scenarios of a sensor operator tasks. Five unique scenarios were created in the simulator, with the primary difference being the difficulty level (3 easy and 2 hard scenarios). Within each difficulty level, the scenarios were randomized. The exact administration of the task is provided in Fig. 1A. During each scenario (referred to as task condition from now on), participants were instructed to complete two synchronous tasks: (1) operate the UAS sensor camera to scan along the designated flight path, and (2) identify and track red civilian bus. Further details related to the simulation and experimental design can be found in^70^.

### Study II: hypercapnic stimuli

Five healthy controls between the ages of 24 and 41 (2 female) voluntarily consented to participate in two University of Pennsylvania IRB approved studies. The research was performed in accordance with relevant regulations. All participants had no history of traumatic brain injury (TBI), pregnancy, and pre-existing disabling neurological or psychiatric disorders. Hypercapnic and normocapnic conditions were administered to each subject via the fixed inspired method using a Douglas Bag^74^. The fixed inspired method delivers room air to the subject at the baseline/normocapnic condition and CO_2_ enriched air during hypercapnic condition, which is kept at 5% CO_2_. The stimulus was provided in a block design manner, where normocapnia and hypercapnia were induced in an alternating manner. Each block was maintained for 60 s, and the experiment was conducted for a total of 7 min (see Fig. 1B). Detailed description of instrumentation set up and how the protocol was administered can be found in^71^.

### fNIRS instrumentation

During both studies, hemodynamic changes within the PFC of the participants were monitored using the fNIR Imager 1200 (fNIR Devices LLC, Potomac, MD). This continuous wave fNIRS system is composed of three parts: a flexible sensor pad that is placed over the forehead to monitor the underlying dorsal and inferior frontal cortical areas; a control box for hardware management; and a computer that runs the data acquisition and visualization software – COBI Studio.

**Figure 2:**
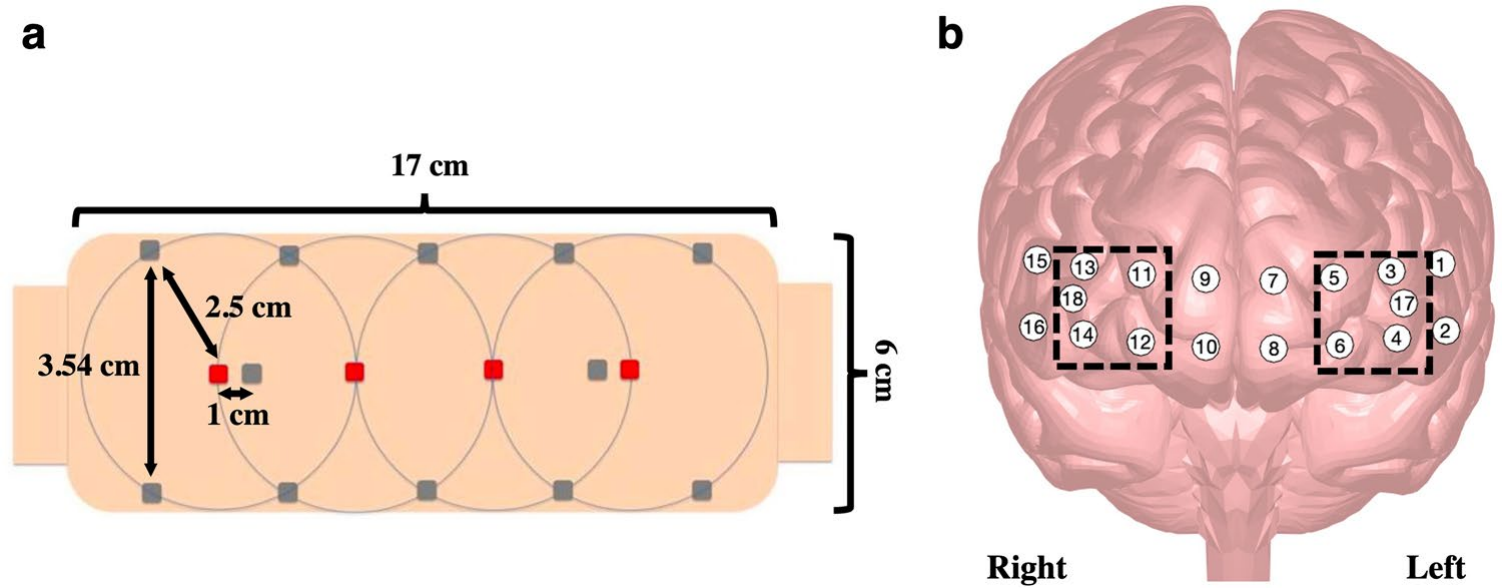
Continuous wave functional Near Infrared Spectroscopy (CW-fNIRS) sensor used. (**a**) Sensor layout, where red squares represent light sources (LEDs), while grey squares represent the photodetectors. (**b**) Location of the 16 long and 2 short source-detector separation channels overlayed on the prefrontal cortex. Black boxes highlight the channels used to calculate measures for right and left middle frontal areas.

The sensor consists of four surface mount light emitting diodes (LED), and twelve silicone photodiodes with integrated trans-impedance preamp housed on a flexible printed circuit board covered with a soft foam for comfort, insulation, durability, flexibility, and safety. The LEDs illuminate light at peak wavelengths of 730 nm and 850 nm, specifically selected to allow spectroscopic conversion without crosstalk^75^. Raw intensity measurements at 730 and 850 nm wavelengths and at dark current condition (when no light is shone) are collected by the system serially from each channel at every 100 ms (10 Hz sampling rate). Ten of the twelve detectors are placed 2.5 cm away from each of the four sources, and the remaining two detectors are placed 1 cm away from the middle two sources (see Fig. 2A). With the given source-detector configuration and sensor placement on the forehead, 16 long SDS and 2 short SDS measurements can be collected from the prefrontal areas of the head (see Fig. 2B).

### Preprocessing of data for artifact removal and hemodynamic conversion

We used Matlab (MathWorks, R2019b) to preprocess and prepare data for statistical analysis. Channels whose raw intensity measures were saturated (> 4500), had high dark current values (> 200), or had high correlation with dark current measurements for each channel (r > 0.7), suggesting poor coupling with the skin, were removed from further analysis^76,77^. Following channel rejection, wavelet-based motion artifact removal (the tuning parameter was set to 0.1) was applied to remove abrupt spikes^61^. Then, the modified Beer-Lambert law (MBLL) was used to calculate the changes in the concentrations of HbO and HbR during the task relative to a baseline ($\Delta {\rm c}^{{{\text{HbO}}}} {\text{ and }} \Delta {\rm c}^{{{\text{HbR}}}} )$ from the intensity measurements (${\text{I}}$) at two wavelengths, using Eqs. (1) and (2).

1 $$\Delta {\text{OD}}_{{\uplambda }} = \log \left( {\frac{{{\text{I}}_{{{\text{baseline}}}} }}{{{\text{I}}_{{{\text{task}}}} }}} \right)$$

2 $$\left[ {\begin{array}{*{20}c} {\Delta {\text{OD}}_{{{\uplambda }1}} } \\ {\Delta {\text{OD}}_{{{\uplambda }2}} } \\ \end{array} } \right] = \left[ {\begin{array}{*{20}c} {{\upvarepsilon }_{{{\uplambda }1}}^{{{\text{HbR}}}} \cdot {\text{d}} \cdot {\text{DPF}}_{{{\uplambda }1}} } & {{\upvarepsilon }_{{{\uplambda }1}}^{{{\text{HbO}}}} \cdot {\text{d}} \cdot {\text{DPF}}_{{{\uplambda }1}} } \\ {{\upvarepsilon }_{{{\uplambda }2}}^{{{\text{HbR}}}} \cdot {\text{d}} \cdot {\text{DPF}}_{{{\uplambda }2}} } & {\varepsilon_{{{\uplambda }2}}^{{{\text{HbO}}}} \cdot {\text{d}} \cdot {\text{DPF}}_{{{\uplambda }2}} } \\ \end{array} } \right]\left[ {\begin{array}{*{20}c} {\Delta {\text{c}}^{{{\text{HbR}}}} } \\ {\Delta {\text{c}}^{{{\text{HbO}}}} } \\ \end{array} } \right]$$

$\Delta {\text{OD}}_{{\uplambda }}$ is change in light attenuation or optical density at a given wavelength (${\uplambda }$) (730 or 850 nm) during a task condition relative to baseline, ε_λ_^X^ (ε_730_^HbO^ = 0.390, ε_730_^HbR^ = 1.102, ε_850_^HbO^ = 1.058, and ε_850_^HbR^ = 0.691) is the wavelength dependent molar extinction coefficient of chromophore X (HbO or HbR), d is the distance between source and detector, DPF_λ_ is the wavelength dependent differential pathlength factor that relates the distance that light travels from the source to the detectors with scattering and absorption effects^78,79^. DPF_λ_ was corrected for age^16^. Spline interpolation method was used on HbO, HbR and total hemoglobin (HbT = HbO + HbR) to correct abrupt signal shifts caused by movement of the sensor or the head^80^. Measurements from channels 3 to 6 and 11 to 14 were averaged and labelled as left and right long SDS regions, respectively. Alternatively channels 17 and 18 were used to represent left and right short SDS regions, respectively. Lastly, to ensure reliable extraction of wavelet coefficients, fNIRS measures were detrended by subtracting a third order polynomial fit and bandpass filtered with cut-off frequencies at 0.009 Hz, and 2 Hz as described in^81^.

### Spectral analysis by wavelet transform

Continuous wavelet transform (CWT) allows for representation of signals in the time–frequency plane. CWT of a signal x(t) can be obtained by using Eq. (3) where ${\text{W}}\left( {{\text{s}},{\text{t}}_{0} } \right)$ is the wavelet transform of x(t), ${\text{t}}_{0}$ is time shift, ${\text{s}}$ is scale, and ${\uppsi }_{{\left( {{\text{s}},{\text{t}}_{0} } \right)}} \left( {\frac{{{\text{t}} - {\text{t}}_{0} }}{{\text{s}}}} \right)$ is the scaled and translated mother wavelet function.

3 $${\text{W}}\left( {{\text{s}},{\text{t}}_{0} } \right) = { }\mathop \smallint \limits_{ - \infty }^{\infty } {\text{x}}\left( {\text{t}} \right).\frac{1}{{\sqrt {\text{s}} }}{\uppsi }_{{\left( {{\text{s}},{\text{t}}_{0} } \right)}} \left( {\frac{{{\text{t}} - {\text{t}}_{0} }}{{\text{s}}}} \right){\text{dt}}$$

Similar to other studies that have investigated cardiovascular dynamics using CWT, the present study used Morlet wavelet as the mother wavelet^29,60^. Morlet wavelet is a Gaussian function, modulated by a sine wave, whose time and frequency domain representation are shown in Eqs. (4) and (5), respectively, where ${\text{f}}_{0}$ is the frequency and time resolution parameter.

4 $${\uppsi }\left( {\text{t}} \right) = { }\frac{1}{{\sqrt {2{\uppi }} }}\left( {{\text{e}}^{{{\text{i}}2{\pi f}_{0} {\text{t}}}} { } - {\text{ e}}^{{{\raise0.7ex\hbox{${ - \left( {2{\pi f}_{0} } \right)^{2} }$} \!\mathord{\left/ {\vphantom {{ - \left( {2{\pi f}_{0} } \right)^{2} } 2}}\right.\kern-\nulldelimiterspace} \!\lower0.7ex\hbox{$2$}}}} } \right){\text{e}}^{{ - {\raise0.7ex\hbox{${{\text{t}}^{2} }$} \!\mathord{\left/ {\vphantom {{{\text{t}}^{2} } 2}}\right.\kern-\nulldelimiterspace} \!\lower0.7ex\hbox{$2$}}}}$$

5 $${\uppsi }\left( {\text{f}} \right) = { }\left( {{\text{e}}^{{ - \frac{1}{2}{ }\left( {2{\pi f}_{0} - {\text{f}}} \right)^{2} }} - {\text{ e}}^{{ - \frac{1}{2}(\left( {2{\pi f}_{0} )^{2} + {\text{f}}^{2} } \right)}} } \right)$$

For Morlet function, the central frequency can be defined as $${\text{f}} = { }{\raise0.7ex\hbox{${{\text{f}}_{0} }$} \!\mathord{\left/ {\vphantom {{{\text{f}}_{0} } {\text{s}}}}\right.\kern-\nulldelimiterspace} \!\lower0.7ex\hbox{${\text{s}}$}}$$. Similar to other studies using wavelet transform, an $${\text{f}}_{0}$$ of 1 was used^29,60^. Furthermore, the frequency limits were set to 0.009 Hz and 2 Hz. This range was chosen to allow for extraction of frequencies associated with: 1) cardiac activity [0.4, 2] Hz; 2) respiratory activity [0.15, 0.4] Hz; 3) myogenic activity [0.06, 0.15] Hz; and 4) VLF activity [0.009, 0.06] Hz^82^. Detailed explanation of the transform, choice of resolution and the toolbox used to perform CWT is described in^81^.

### Statistics

In order to statistically evaluate wavelet coefficients across bands, tissue layers, hemispheres, and stimuli, we used quantitative measures established by^60^. The first quantitative measure is the time-averaged energy density (${\upvarepsilon }$) within a given frequency band, which was calculated using Eq. (6) where ${\text{f}}_{1} ,\;{\text{f}}_{2}$ are minimum and maximum frequencies associated with each band (Ex. Cardiac: ${\text{f}}_{{1{ }}} = { }0.4{\text{ Hz }},\;{\text{f}}_{2} = 2{\text{ Hz}}$).

6 $${\upvarepsilon }\left( {{\text{f}}_{1} ,{\text{f}}_{2} } \right) = { }\frac{1}{{\text{t}}}\mathop \smallint \limits_{0}^{{\text{t}}} \mathop \smallint \limits_{{{\raise0.7ex\hbox{$1$} \!\mathord{\left/ {\vphantom {1 {2{\pi f}_{2} }}}\right.\kern-\nulldelimiterspace} \!\lower0.7ex\hbox{${2{\pi f}_{2} }$}}}}^{{{\raise0.7ex\hbox{$1$} \!\mathord{\left/ {\vphantom {1 {2{\pi f}_{1} }}}\right.\kern-\nulldelimiterspace} \!\lower0.7ex\hbox{${2{\pi f}_{1} }$}}}} \frac{1}{{s^{2} }}\left| {{\text{W}}\left( {{\text{s}},{\text{t}}} \right)} \right|^{2} {\text{dsdt}}$$

Overall magnitude of ${\upvarepsilon }$ is sensitive to subject-dependent factors, such as activation in hemisphere with task condition. These effects may lead to an underestimation of band and tissue layer effects. Therefore, to mitigate these effects, we calculated relative energy density (${\text{rel}}\upvarepsilon$) using Eq. (7) where ${\upvarepsilon }_{{{\text{total }}\;{\text{average}}}}$ is the average energy density across the entire frequency spectrum (0.009 Hz to 2 Hz).

7 $${\text{rel}}\upvarepsilon \left( {{\text{f}}_{1} ,{\text{f}}_{2} } \right) = \frac{{{\upvarepsilon }\left( {{\text{f}}_{1} ,{\text{f}}_{2} } \right)}}{{{\upvarepsilon }_{{{\text{total}}\;{\text{average}}}} }}$$

**Figure 3:**
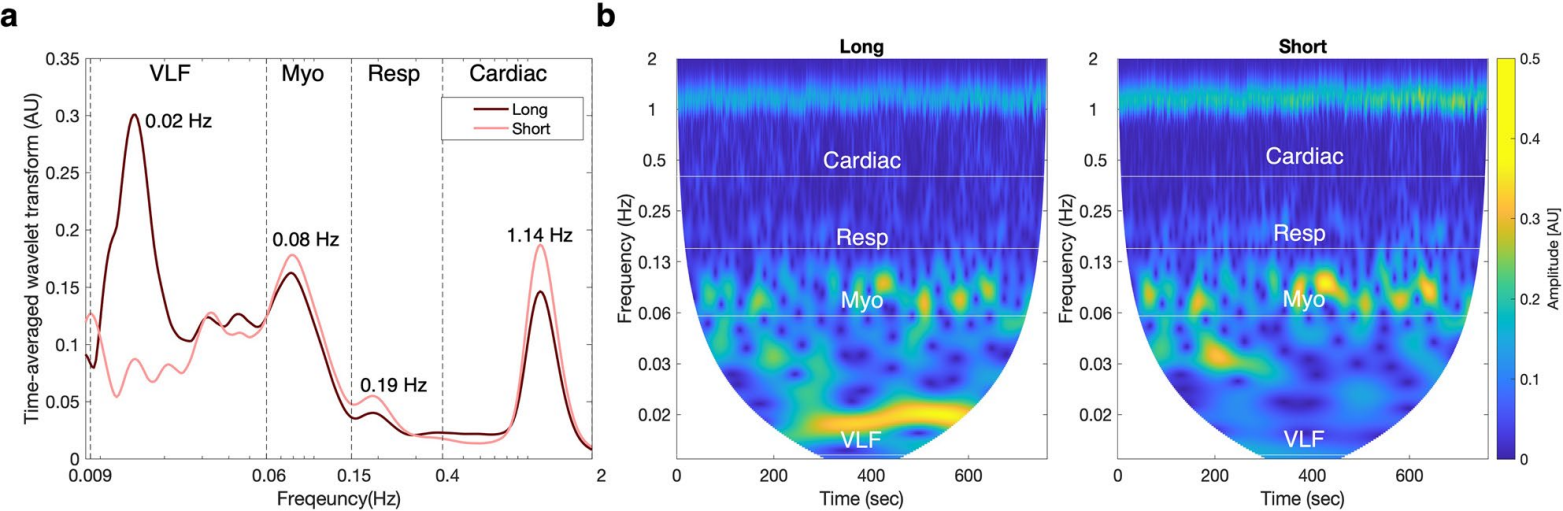
Continuous wavelet transforms of HbO signals from right-middle frontal area of long and short SDS measurements taken during cognitive task for one representative subject. (**a**) Changes in amplitude or absolute value of the wavelet coefficients as a function of frequency. (**b**) Changes in amplitude as a function of time and frequency. Dashed vertical lines in A and horizontal lines in B represent frequency intervals for very low frequency (VLF), myogenic (Myo), respiratory (Resp) and cardiac bands. Figure generated using custom code in Matlab (MathWorks, R2019b).

An example of changes in time averaged wavelet coefficients across 0.009 to 2 Hz of long- and short-SDS measurements taken from one subject is shown in Fig. 3A. Strong activity can be seen in the VLF band, followed by myogenic, cardiac, and respiratory bands. The magnitude in the VLF band was greater in long-SDS measurements, while all other bands had greater magnitudes in short-SDS measurements. Apart from the VLF band, local maxima appear to occur at the same frequencies in both long- and short-SDS measurements. Specifically, oscillations appear at 1.14 Hz, 0.19 Hz, and 0.08 Hz of the cardiac, respiration and myogenic bands, respectively. In the VLF band a large peak emerged at 0.02 Hz in the long-SDS measurements, but not in short-SDS measurements. The changing periodicity of the local maxima is visualized in the plotted scalogram (see Fig. 3B). The results indicate that cardiac and VLF peaks remained stationary for the most part, while respiratory and myogenic peaks varied with time.

We used R (R Core Team, 2019), *lme4, lmerTest* and *emmeans* to perform linear mixed effects (LME) analyses^83–85^. Model development investigated the main and interaction effects of band (VLF, myogenic, respiratory, cardiac), SDS (long and short), and hemisphere (left and right) on ${\upvarepsilon }$ and ${\text{rel}}\upvarepsilon$ per biomarker (HbO, HbR and HbT) for each stimulus (cognitive and hypercapnic). During evaluation of cognitive stimulus, condition (Easy1 (E1), Easy2 (E2), Easy3 (E3), Hard1 (H1), and Hard2 (H2)) was added as another factor to investigate task-evoked systemic and neuronal effects. Model terms were built on a-priori knowledge and selected based on backward elimination of non-significant predictors from a saturated model^84^. The final model chosen for cognitive and hypercapnic stimuli are coded using *lme4* syntax from R and are shown in Eqs. (8) and (9), respectively. Significance of fixed effect terms were evaluated using likelihood ratio tests, where the full effects model was compared against a model without the effect in question (E.g., 1 + Band + (1|Participant) vs 1 + (1|Participant) when evaluating significant effect of Band). Post hoc analysis was conducted to evaluate differences between levels per each model term. Seven planned comparisons were performed when evaluating both stimuli, where three were for “Band” (VLF vs myogenic, respiratory, or cardiac) and four were for “Band: SDS” (e.g., long vs short from VLF). For cognitive stimulus, an additional 56 planned comparisons were performed, where eight were for “Band: SDS : Hemisphere” (e.g. left vs right from VLF of short), and 48 were for “Band : SDS : Condition” (i.e., E1 vs E2, E1 vs E3, H1 vs H2, E1 vs H1, E2 vs H2, and E3 vs H1 from VLF of short) terms. Example of code and resulting output of the likelihood ratio and post hoc tests used are shown in Supplemental Table 1.

8 $$1 + {\text{Band}} + {\text{Band}}:{\text{SDS}} + {\text{Band}}:{\text{SDS}}:{\text{Hemisphere}} + {\text{Band}}:{\text{SDS}}:{\text{Condition}} + \left( {1{\text{|SubjectID}}} \right)$$

9 $$1 + {\text{Band}} + {\text{Band}}:{\text{SDS}} + \left( {1{\text{|SubjectID}}} \right)$$

Homogeneity of variance and normality tests were conducted using visual inspection of residuals and Q–Q plots for all models. If model predictions showed heteroscedasticity or a non-normal distribution, then log10 transformations were performed on the response variables. However, model estimates ($\beta$) and confidence intervals ($CI$) were back transformed to original units. Satterthwaite approximation of degrees of freedom was used in post hoc analyses^86^. For all statistical analyses, the level of significance was set at ${\upalpha }$ = 0.05. Adjustments using false discovery rate (FDR) were made on p-values to account for Type I error inflation. Lastly, Cohen’s d was calculated per post hoc comparison to examine effect sizes^87^. Cohen’s d of 0.2, 0.5 and > 0.8 reflect small, medium and large effects, respectively. Coding used to calculate these effect sizes are shown in Supplemental Table 1.

## Results

### Study I: cognitive stimulus

**Figure 4:**
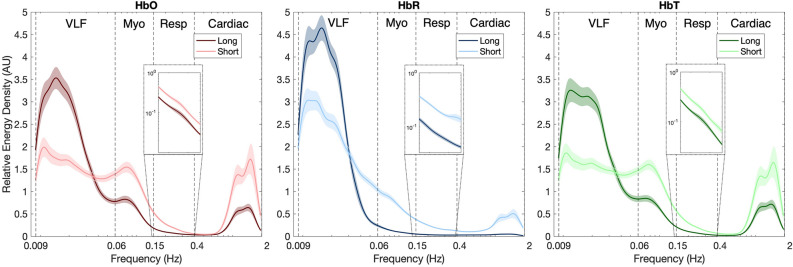
Differences in relative energy density ($rel{\upvarepsilon })$ between long- and short- source detector separation (SDS) measurements per biomarker. Colored lines represent mean of the data (channels, conditions and subjects), while associated shaded portions represent standard error of mean. Dashed black lines represent beginning and ending of very low frequency (VLF), myogenic (Myo), respiratory (Resp) and cardiac bands. The embedded plot represents log-transformed $rel{\upvarepsilon }$ values of respiratory band (0.15 to 0.4 Hz). Figure generated using custom code in Matlab (MathWorks, R2019b).

Figure 4 shows the comparison of $rel{\upvarepsilon }$ from short- and long-SDS channels across all subjects for each biomarker. Over the frequency range of 0.009 to 2 Hz, both SDSs presented similar average wavelet coefficients with noticeable local maxima at ~ 1 to 1.5 Hz, ~ 0.25 Hz, ~ 0.08 and ~ 0.01 to 0.02 Hz in cardiac, respiratory, myogenic and VLF bands, respectively. These local maxima were visible in HbO and HbT, but were not visible in HbR, especially those associated with myogenic and respiration activity.

Statistical evaluation revealed significant effects of band per biomarker for ${\text{rel}}\upvarepsilon$ (HbO: ${\upchi }^{2}$(3) = 1178.0, *p* < 0.001; HbR: $\chi^{2}$(3) = 1178.1, *p* < 0.001; HbT: $\chi^{2}$(3) = 1133.1, *p* < 0.001) and ${\upvarepsilon }$ (HbO: ${\upchi }^{2}$(3) = 979.8, *p* < 0.001; HbR: $\chi^{2}$(3) = 1170.0, *p* < 0.001; HbT: $\chi^{2}$(3) = 890.7, *p* < 0.001). Regardless of the biomarker, power was contained primarily in VLF band, followed by myogenic, cardiac, and respiratory bands, as shown by decreasing ${\upbeta }$ values per biomarker (see Estimate Band column in Table 1). Post hoc comparisons between bands revealed significant differences between VLF and other bands (see Post-hoc Band column of Table 1). Specifically, the respiratory band had the largest deviation from the VLF band (HbO: *p* < 0.001, d = 4.89; HbR: *p* < 0.001, d = 6.39; HbT: *p* < 0.001, d = 4.56), followed by the cardiac (HbO: *p* < 0.001, d = 3.09; HbR: *p* < 0.001, d = 6.36; HbT: *p* < 0.001, d = 2.95) and myogenic bands (HbO: *p* < 0.001, d = 1.64; HbR: *p* < 0.001, d = 4.21; HbT: *p* < 0.001, d = 1.46). HbR had the largest effects across the four bands, followed by HbO and HbT.

**Table 1:** Differences in relative energy density ($rel{\upvarepsilon }$) values between bands and between SDS measurements for each band per biomarker of cognitive stimulus.

Biomarker	Band	Estimate band	Post-hoc band		Estimate SDS		Post-hoc SDS	
					Short	Long		
		$\beta$[95% $CI$]	$adj. p$	$d$	$\beta$[95% $CI$]	$\beta$[95% $CI$]	$adj. p$	$d$
ΔHbO	VLF	1.82 [1.63, 2.04]	n/a	n/a	1.48 [1.30, 1.68]	2.25 [1.98, 2.57]	** < 0.001**	− 0.74
	Myo	0.72 [0.65, 0.81]	** < 0.001**	1.64	1.09 [0.96, 1.24]	0.48 [0.42, 0.55]	** < 0.001**	1.45
	Resp	0.11 [0.10, 0.13]	** < 0.001**	4.89	0.19 [0.16, 0.21]	0.07 [0.06, 0.08]	** < 0.001**	1.73
	Cardiac	0.32 [0.28, 0.36]	** < 0.001**	3.09	0.47 [0.41, 0.53]	0.22 [0.19, 0.25]	** < 0.001**	1.36
ΔHbR	VLF	2.41 [2.12, 2.73]	n/a	n/a	2.14 [1.85, 2.46]	2.71 [2.35, 3.12]	**0.002**	− 0.43
	Myo	0.24 [0.21, 0.27]	** < 0.001**	4.21	0.63 [0.54, 0.72]	0.09 [0.08, 0.10]	** < 0.001**	3.53
	Resp	0.07 [0.06, 0.08]	** < 0.001**	6.39	0.18 [0.16, 0.21]	0.03 [0.02, 0.03]	** < 0.001**	3.35
	Cardiac	0.07 [0.06, 0.08]	** < 0.001**	6.36	0.17 [0.15, 0.20]	0.03 [0.03, 0.04]	** < 0.001**	3.07
ΔHbT	VLF	1.80 [1.60, 2.01]	n/a	n/a	1.46 [1.28, 1.67]	2.21 [1.94, 2.53]	** < 0.001**	− 0.71
	Myo	0.76 [0.68, 0.85]	** < 0.001**	1.46	1.13 [0.98, 1.29]	0.52 [0.45, 0.59]	** < 0.001**	1.32
	Resp	0.12 [0.11, 0.14]	** < 0.001**	4.56	0.20 [0.18, 0.23]	0.08 [0.07, 0.09]	** < 0.001**	1.69
	Cardiac	0.32 [0.28, 0.36]	** < 0.001**	2.95	0.44 [0.38, 0.50]	0.23 [0.20, 0.26]	** < 0.001**	1.08

$\beta$ Estimate, $CI$ confidence interval;$adj.p$ FDR adjusted *p* values with bolded values representing significance at α < 0.05, $d$ Cohen’s d. In post-hoc band column, the metrics represent differences between very low frequency (VLF) and other bands (e.g., Myogenic – VLF), where positive $d$ means that the effect was more pronounced in VLF band. Alternatively, in post-hoc SDS column the metrics represent differences between short- and long-SDS measurements, where negative $d$ values mean the effect was more pronounced in long-SDS measurements.

SDS was a significant predictor per biomarker of ${\text{rel}}\upvarepsilon$ (HbO: $\chi^{2}$(4) = 379.4, *p* < 0.001; HbR: $\chi^{2}$(4) = 1109.9, *p* < 0.001; HbT: $\chi^{2}$ (4) = 327.8, *p* < 0.00) and ${\upvarepsilon }$ (HbO: $\chi^{2}$(4) = 163.0, *p* < 0.001; HbR: $\chi^{2}$(4) = 354.8, *p* < 0.001; HbT: $\chi^{2}$ (4) = 204.6, *p* < 0.001). Long-SDS measurements were dominated by VLF activity, while short-SDS measurements were primarily influenced by systemic activity, as reflected by the negative Cohen’s $d$ values in VLF band, and the positive in systemic bands (see Post-hoc SDS column of Table 1). Regardless of the biomarker, differences were largest in the respiratory (HbO: *p* < 0.001, d = 1.73; HbR: *p* < 0.001, d = 3.35; HbT: *p* < 0.001, d = 1.69), followed by cardiac (HbO: *p* < 0.001, d = 1.36; HbR: *p* < 0.001, d = 3.07; HbT: *p* < 0.001, d = 1.08), myogenic (HbO: *p* < 0.001, d = 1.45; HbR: *p* < 0.001, d = 3.53; HbT: *p* < 0.001, d = 1.32) and VLF (HbO: *p* < 0.001, d = − 0.74; HbR: *p* = 0.002, d = -0.43; HbT: *p* < 0.001, d = − 0.72) bands. The size of the effect for the VLF band was largest in HbO, followed by HbT and HbR. In contrast, systemic activities had largest effects on HbR, followed by HbT and HbO.

The three way interaction among band, SDS and hemisphere was significant across all biomarkers for ${\text{rel}}\upvarepsilon$ (HbO: $\chi^{2}$(8) = 44.0, *p* < 0.001; HbR: ${\upchi }^{2}$(8) = 47.8, p < 0.001; HbT:$\chi^{2}$(8) = 35.4, *p* < 0.001) and ${\upvarepsilon }$ (HbO: $\chi^{2}$(8) = 31.8, p < 0.001; HbR: ${\upchi }^{2}$(8) = 41.1, *p* < 0.001; HbT:$\chi^{2}$(8) = 22.5, p = 0.004). As displayed in Supplemental Table 2, the ${\upvarepsilon }$ values from VLF band of long-SDS measurements showed considerable differences between right- and left-middle frontal areas for HbO (*p* = 0.016, adjusted *p* = 0.068, d = − 0.64) and HbR (*p* = 0.018, adjusted *p* = 0.142, d = − 0.72), with HbR revealing a larger medium effect. The negative Cohen’s $d$ values suggests that the right-middle frontal area had dominant activity. In systemic bands, only cardiac band displayed considerable differences between the two hemispheres. These differences were seen in the short-SDS measurements of HbO (*p* = 0.017, adjusted *p* = 0.068, d = 0.63) and HbT (*p* = 0.020, adjusted *p* = 0.068, adjusted *p* = 0.163, d = 0.58), and long-SDS measurements of HbR (*p* = 0.037, adjusted *p* = 0.149, d = − 0.53). The effects associated were medium in size, with HbO exhibiting the largest effect and being dominant in right-middle frontal areas.

**Figure 5:**
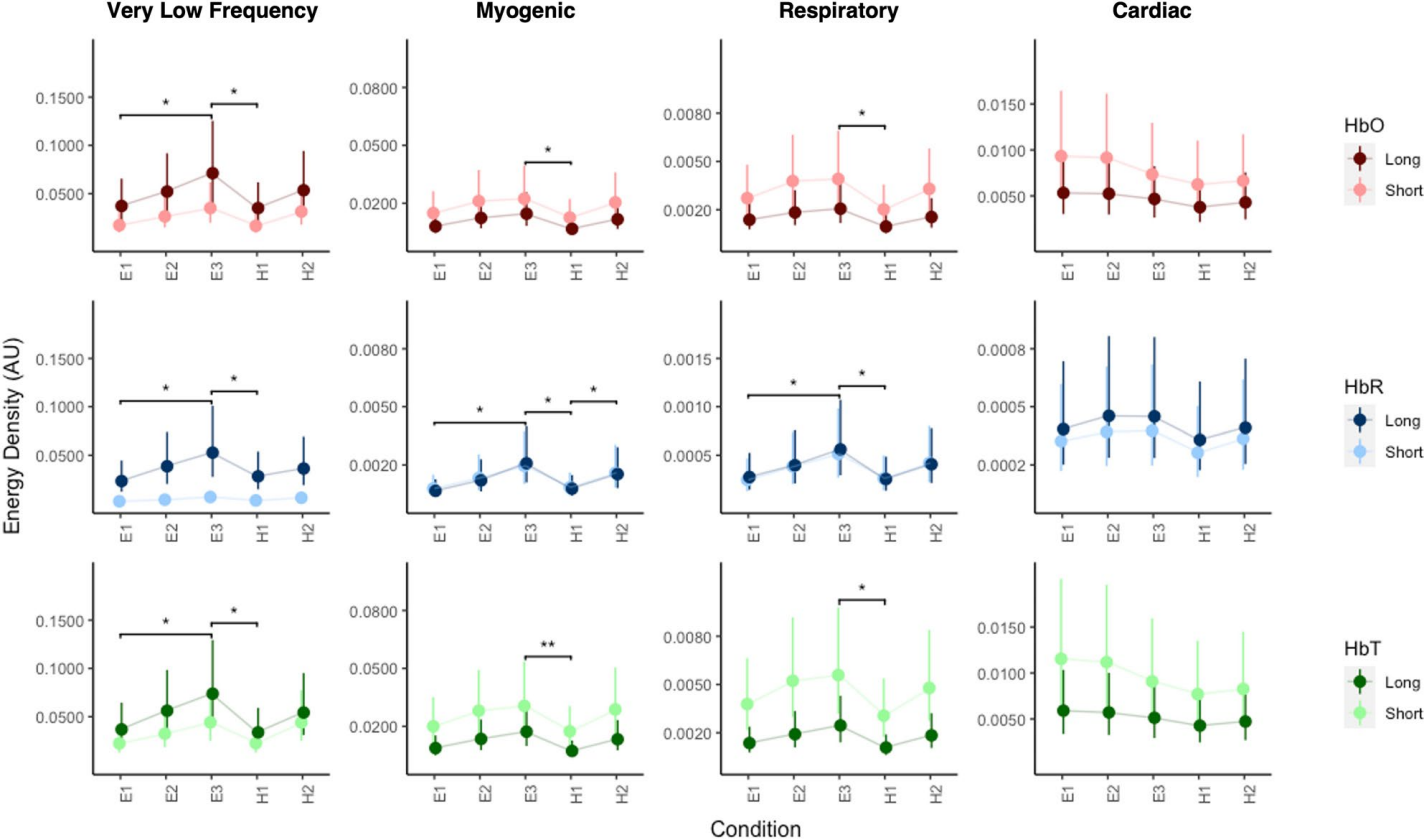
Effect of condition on ${\upvarepsilon }$ per SDS measurement, band and biomarker. Dots represent mixed model estimates, while lines represent confidence intervals. **p < 0.01, *p < 0.05. Significance bars reflect p values that were not adjusted using FDR correction. Only conditions pairs that were significant in both SDS measurements are shown here. Figure generated using custom code in R (R Core Team, 2019).

Interaction between band, SDS and condition was significant across all biomarkers for ${\text{rel}}\upvarepsilon$ (HbO: $\chi^{2}$(32) = 69.9, *p* < 0.001; HbR: $\chi^{2}$(32) = 86.1, *p* < 0.001; HbT:$\chi^{2}$(32) = 67.9, *p* < 0.001) and ${\upvarepsilon }$ (HbO: $\chi^{2}$(32) = 117.2, *p* < 0.001; HbR: $\chi^{2}$(32) = 153.3, *p* < 0.001; HbT:$\chi^{2}$(32) = 116.6, *p* < 0.001). ${\upvarepsilon }$ increased across conditions of same difficulty level in VLF, myogenic and respiratory bands for all biomarkers (see Fig. 5 and Supplemental Table 3). Alternatively, ${\upvarepsilon }$ decreased in cardiac bands of HbO and HbT biomarkers and increased in HbR. Differences between recordings of the third and last easy (E3) and the first hard task condition (H1) was significant across all SDS measurements and biomarkers of VLF, myogenic and respiratory bands, with E3 having higher values. No significant differences were found between conditions of different difficulty (i.e., E1 vs H1 or E2 vs H2). Differences within the same difficulty conditions varied with band and SDS measurement. In the VLF band, E1 vs E3 was considerably different for both long (HbO: *p* = 0.022, adjusted *p* = 0.177, d = − 0.91; HbR: *p* = 0.022, adjusted *p* = 0.181, d = − 1.05; HbT: *p* = 0.019, adjusted *p* = 0.189, d = − 0.94) and short SDS measurements (HbO: *p* = 0.017, adjusted *p* = 0.177, d = − 0.99; HbR: *p* = 0.013, adjusted *p* = 0.181, d = − 1.36; HbT: *p* = 0.020, adjusted *p* = 0.189, d = − 0.92), while H1 vs H2 was considerably different only for short-SDS measurements of all biomarkers (HbO: *p* = 0.023, adjusted *p* = 0.177, d = − 0.89; HbR: *p* = 0.044, adjusted *p* = 0.193, d = − 0.79; HbT: *p* = 0.021, adjusted *p* = 0.189, d = − 0.91). In the myogenic band, E1 vs E3 (HbO: *p* = 0.029, adjusted *p* = 0.177, d = − 0.83; HbR: *p* = 0.011, adjusted *p* = 0.181, d = -1.49; HbT: *p* = 0.021, adjusted *p* = 0.189, d = − 0.92) and H1 vs H2 (HbO: p = 0.037, adjusted *p* = 0.195, d = − 0.77; HbR: *p* = 0.034, adjusted *p* = 0.181, d = − 0.87; HbT: *p* = 0.029, adjusted *p* = 0.194, d = − 0.82) was considerably different for all biomarkers of long-SDS measurements, however both condition pairs (E1 vs E3: *p* = 0.017, adjusted *p* = 0.181, d = − 1.18; H1 vs H2: *p* = 0.039, adjusted *p* = 0.193, d = − 0.82) were only considerably different for HbR of short-SDS measurements. In the respiratory band only E1 vs E3 of HbR (Short: *p* = 0.030, adjusted p = 0.181, d = − 0.92; Long: *p* = 0.027, adjusted *p* = 0.181, d = − 0.95) was considerably different.

### Study II: hypercapnic stimulus

**Figure 6:**
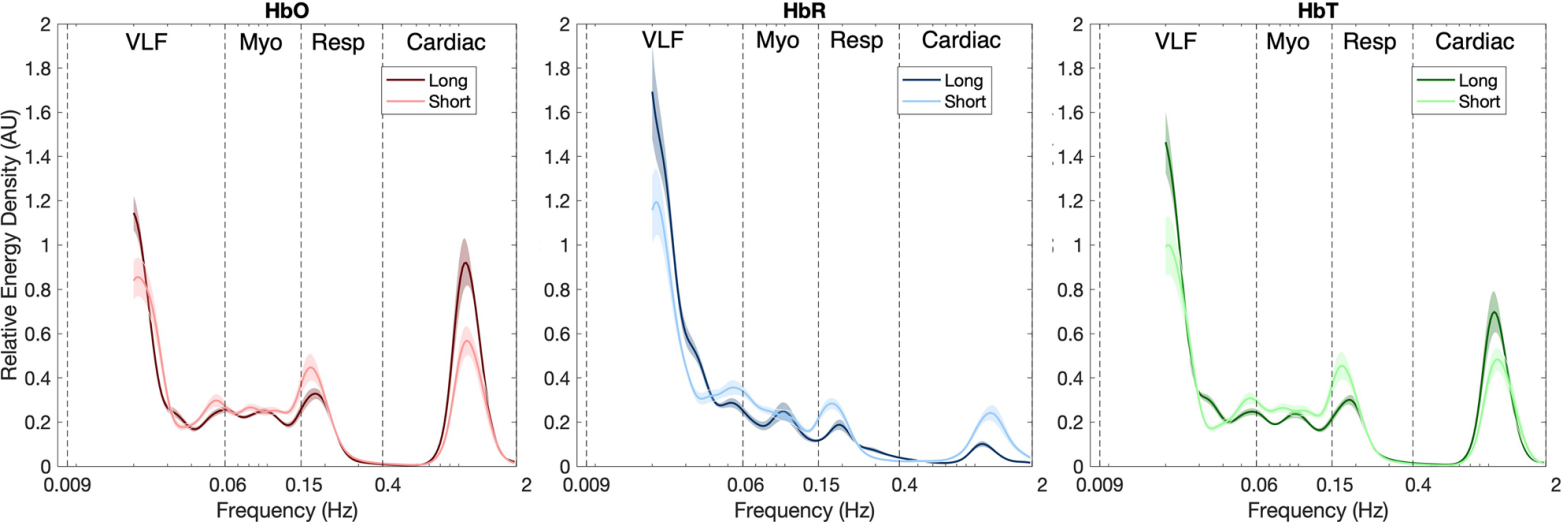
Differences in relative energy density ($rel{\upvarepsilon })$ between long- and short- source detector separation (SDS) measurements per biomarker during hypercapnic stimulus. Colored lines represent mean of the data (channels and subjects), while associated shaded portions represent standard error of mean. Dashed black lines represent beginning and ending of very low frequency (VLF), myogenic (Myo), respiratory (Resp) and cardiac bands. Figure generated using custom code in Matlab (MathWorks, R2019b).

Figure 6 shows average ${\text{rel}}\upvarepsilon$ of long- and short-SDS measurements per biomarker, when subjects were administered with hypercapnic stimulus. Noticeable local maxima at ~ 1.2 Hz, ~ 0.2 Hz, and ~ 0.1 can be seen in cardiac, respiratory and VLF bands of HbO and HbT, respectively. Alternatively, multiple local maxima of small amplitude can be seen in myogenic band.

Statistical evaluation of the effect of band revealed to be significant across all biomarkers of ${\text{rel}}\upvarepsilon$ (HbO: ${\upchi }^{2}$(3) = 25.9, p < 0.001; HbR: $\chi^{2}$(3) = 77.1, *p* < 0.001; HbT:$\chi^{2}$(3) = 31.5, *p* < 0.001) and ${\upvarepsilon }$ (HbO: $\chi^{2}$(3) = 20.3, *p* < 0.001; HbR: $\chi^{2}$(3) = 63.8, *p* < 0.001; HbT:$\chi^{2}$(3) = 24.2, *p* < 0.001). As indicated by decreasing ${\upbeta }$ values (see Estimate Band column in Table 2), ${\text{rel}}\upvarepsilon$ was concentrated in VLF band, followed by myogenic, respiratory, and cardiac band for HbR and HbT biomarker. In HbO, ${\text{rel}}\upvarepsilon$ was concentrated in the VLF band, followed by myogenic, cardiac and respiratory bands. Post hoc comparisons between bands revealed significant differences between VLF and other bands, except for VLF and myogenic band (see Post-hoc Band column of Table 2). Specifically, the VLF band deviated most from cardiac band (HbO: *p* = 0.001, d = 1.58; HbR: *p* < 0.001, d = 4.03; HbT: *p* = 0.001, d = 4.01), followed by respiratory (HbO: *p* = 0.001, d = 1.70; HbR: *p* < 0.001, d = 3.00; HbT: *p* = 0.002, d = 2.99) and myogenic (HbO: *p* = 0.097, d = 0.64; HbR: *p* < 0.001, d = 1.82; HbT: *p* = 0.044, d = 1.81) bands. Overall, HbR displayed the largest differences, followed by HbT and HbO.

**Table 2:** Differences in relative energy density ($rel{\upvarepsilon }$) values between bands and between SDS measurements for each band per biomarker of hypercapnic stimulus.

Biomarker	Band	Estimate band	Post-hoc band		Estimate SDS			Post-Hoc SDS		
					Short	Long				
		$\beta$[95% $CI$]	$adj. p$	$d$	$\beta$[95% $CI$]		$\beta$[95% $CI$]		$adj.p$	$d$
ΔHbO	VLF	1.45 [1.07, 1.96]	n/a	n/a	1.42 [0.90, 2.24]		1.49 [1.01, 2.18]		0.874	− 0.08
	Myo	0.98 [0.73, 1.33]	0.097	0.64	1.13 [0.72, 1.79]		0.86 [0.58, 1.26]		0.494	0.46
	Resp	0.52 [0.39, 0.71]	**0.001**	1.70	0.61 [0.39, 0.96]		0.45 [0.31, 0.66]		0.494	0.51
	Cardiac	0.56 [0.41, 0.76]	**0.001**	1.58	0.83 [0.24, 0.60]		0.38 [0.56, 1.21]		0.066	-1.28
ΔHbR	VLF	2.55 [1.79, 3.62]	n/a	n/a	2.00 [1.17, 3.42]		1.43 [0.87, 2.34]		0.173	− 0.68
	Myo	0.70 [0.49, 0.99]	** < 0.001**	1.82	1.03 [0.60, 1.76]		1.13 [0.69, 1.86]		0.102	1.08
	Resp	0.30 [0.21, 0.43]	** < 0.001**	3.00	0.48 [0.28, 0.83]		0.60 [0.37, 0.99]		0.102	1.33
	Cardiac	0.15 [0.10, 0.21]	** < 0.001**	4.03	0.21 [0.12, 0.36]		0.31 [0.19, 0.51]		0.102	1.04
ΔHbT	VLF	1.59 [1.13, 2.23]	n/a	n/a	1.43 [0.87, 2.34]		1.77 [1.16, 2.70]		0.513	− 0.34
	Myo	0.94 [0.67, 1.33]	**0.044**	1.81	1.13 [0.69, 1.86]		0.79 [0.52, 1.20]		0.513	0.57
	Resp	0.54 [0.38, 0.76]	**0.002**	2.99	0.60 [0.37, 0.99]		0.49 [0.32, 0.74]		0.513	0.33
	Cardiac	0.44 [0.31, 0.62]	**0.001**	4.01	0.31 [0.19, 0.51]		0.62 [0.41, 0.94]		0.174	− 1.06

${\upbeta }$ Estimate, $CI$ confidence interval,$adj.p$ FDR adjusted *p* values with bolded values representing significance at α < 0.05; $d$ Cohen’s d. In post-hoc band column, the metrics represent differences between very low frequency (VLF) and other bands (e.g., Myogenic – VLF), where positive $d$ means that the effect was more pronounced in VLF band. Alternatively, in post-hoc SDS column the metrics represent differences between short- and long-SDS measurements, where negative $d$ values mean the effect was more pronounced in long-SDS measurements.

SDS and band interaction on ${\text{rel}}\upvarepsilon$ was a significant factor only for HbR ($\chi^{2}$(4) = 18.20, p = 0.001) and all biomarkers of ${\upvarepsilon }$ (HbO: $\chi^{2}$(4) = 15.59, *p* = 0.001; HbR: $\chi^{2}$(4) = 34.20, *p* < 0.001; HbT: $\chi^{2}$(4) = 21.45, *p* < 0.001). Post hoc analysis between SDS revealed considerable differences only in ${\text{rel}}\upvarepsilon$ for respiratory band of HbR (*p* = 0.034, adjusted *p* = 0.102, d = 1.33) and cardiac band of HbO (*p* = 0.017, adjusted *p* = 0.066, d = − 1.28) and HbT (*p* = 0.044, adjusted *p* = 0.174, d = − 1.06) when *p* was not adjusted for multiple comparisons (see Post-hoc SDS column of Table 2). The respiratory activity was dominant in long-SDS measurements, while cardiac activity was leading in short-SDS measurements.

## Discussion

In this study, we utilized wavelet transform analysis and implemented multiple long- and short- source-detector separation (SDS) channels to evaluate and quantify the contribution of fNIRS signal components with respect to physiological origins, tissue layers and hemispheres during cognitive and hypercapnic stimuli. Overall, our results indicate that fNIRS signal components have unique temporal, spatial and frequency responses to different types of stimuli. The following discussion of these results begins by identifying physiological factors affecting fNIRS signals, followed by how much these factors contribute to various fNIRS markers, and how their contributions vary axially, laterally, and temporally with different stimuli.

Characteristic frequencies were observed on average at around 0.02, 0.09, 0.20 and 1.20 Hz (see Figs. 4 and 6). These detected peaks have appeared at similar frequencies in other fNIRS, fMRI and laser Doppler studies, and have been reported to be due to cardiac, respiratory, myogenic, neuronal and partial arterial carbon dioxide (PaCO_2_) activities^21,88,89^. Specifically, peaks at 0.02 Hz have been attributed to nitric oxide (NO)-related endothelial activity. During increased metabolic demand, release of NO leads to vasodilation of cerebral vessels to allow for constant and optimal nutrient and oxygen supply as well as effective removal of waste products such as CO_2_. Although vasodilation does not occur solely due to NO, studies have reported that NO is responsible for at least 61% of CBF increase^90^. During hypercapnic stimuli, neuronal activity is minimal, therefore the activity within this band can be attributed directly to PaCO_2_-related changes. This change is commonly referred to as cerebrovascular reactivity (CVR)^13,91^. Alternatively, during a cognitive task, since the dilation is being induced by increased neuronal activity, the phenomenon is termed neurovascular coupling (NVC)^92^. However, a cognitive task may elicit both PaCO_2_ and neuronal activity related vasodilation^24^. In fact, PaCO_2_-related effects may contribute to at least 1.5% increase in fNIRS biomarkers when cognitive tasks involving out-loud speech or those requiring mental thought are performed^54^. Since the cognitive task we employed does not require internal or external conversation, we assume that the effects of PaCO_2_ were minimal and that changes reflected are solely due to NVC. Local maxima at ~ 0.09 Hz have been reported to occur due to a combination of Mayer waves and vasomotion. Mayer waves are associated with arterial pressure and sympathetic response, while vasomotion is defined as the oscillation in the tone of blood vessels that is independent of respiration, pulsation and neuronal activity^21,88,93^. Due to high correlation and coherence between vasomotion and Mayer waves, the exact physiological phenomenon behind the observed peaks is difficult to isolate^19^. Peak frequencies at ~ 0.20 Hz and ~ 1.20 Hz correspond to respiration rate and heart rate, respectively^21,29,88,89^.

Contribution of each of the aforementioned factors to the overall fNIRS signal varied with stimuli. Specifically, during cognitive stimulus the VLF band had the highest activity followed by myogenic, cardiac, and respiratory bands (see Table 1). Alternatively, during hypercapnic stimulus, activity was highest in the VLF band followed by cardiac, respiratory, and myogenic bands (see Table 2). Observed contributions of VLF band during both stimuli are in agreement with other spectral analysis studies^29,64,66–69^, and form the physiological foundation behind fNIRS usage to study NVC and CVR. However, the effect size associated with this band was equal in hypercapnic (HbO: d = 0.58; HbR: d = 0.97; HbT: d = 0.55) and cognitive tasks (HbO: d = 0.59; HbR: d = 1.27; HbT: d = 0.55). This finding is in conflict with a transcranial doppler study, which reported that CO_2_ increased cerebral blood flow velocity (CBFV) by 25% while NVC only increased it by 5%^94^. On the other hand, the study utilizing BOLD-fMRI and CBF-ASL, reported comparable increases in measures due to a cognitive task (complex Stroop) and hypercapnic tasks in a young cohort similar to the findings of our study^95^. Differences in myogenic band contributions to fNIRS signals based on stimuli administered are likely due to Mayer waves being attenuated during hypercapnia, but not during cognitive tasks^22^. In fact, in cognitive studies, contributions due to myogenic activity have been shown to reduce the accuracy of extracted neuronal related hemodynamic effects by at least threefold^82^. The respiratory peak had the weakest presence in cognitive task and a noticeable presence in the hypercapnic task. Such changes in respiration activity during cognitive and hypercapnic tasks on fNIRS signals have been reported previously^22^. During the hypercapnic stimulus, increased PaCO_2_ levels stimulate central chemoreceptors to increase the rate and depth of breathing in order to return CO_2_ concentrations to normal resting levels^96^. Alternatively, during functional activation as evoked by cognitive tasks, changes in PaCO_2_ are typically not as drastic, therefore not stimulating chemoreceptors and resulting in weaker respiration activity^22^. Cardiac peaks had the second largest contribution to fNIRS signal during hypercapnic stimuli, but less so in cognitive task. This may be due to the cardio-respiratory coupling being stronger during hypercapnia stimuli than during cognitive stimuli, however further research needs to be conducted to investigate the reasoning behind this finding.

Systemic activity dominated short-SDS measurements, while neuronal-related activity dominated long-SDS measurements in the cognitive task (see Post-hoc SDS columns of Table 1). Such differences were not significant in the hypercapnic task (see Post-hoc SDS columns of Table 2), however trends observed in Fig. 6, indicate that respiratory activity was more dominant in short-SDS measurements, while PaCO_2_ and cardiac activity were stronger in long-SDS measurements. Magnitude of the activity within the VLF band from short-SDS measurements was not negligible for either stimulus. In fact, the band power was greater than that of other systemic bands in both stimuli (see Estimate short-SDS columns of Tables 1 and 2). Furthermore, the VLF band power from long-SDS measurements were greater than that of short-SDS measurements (see Post-hoc SDS columns of Table 1) in the cognitive task but did not differ in the hypercapnic task (see Post-hoc SDS columns of Table 2). Studies have indicated that such activity is likely due to scalp blood flow (SBF)^34–41^. Based on this, the results in this study indicate that measurements taken from long SDS channels reflect a combination of hemodynamic changes associated with NVC and SBF in the cognitive task and strictly SBF in the hypercapnic task. The results from the cognitive task are in agreement with other studies, where activity within VLF band of short-SDS channels have been reported to not only share similar spectral characteristics as that of long-SDS channels, but also contribute at least 80% of the signal detected by at the long-SDS channels^22,33^. However, presence of SBF during the hypercapnic task is surprising, since studies have reported that PaCO_2_ does not induce SBF changes^23,97^. A possible explanation underlying such findings may be that SBF changes in extracerebral layers are temporally delayed, rather than minimal or not present^98,99^. In cognitive tasks, systemic activities such as myogenic, respiratory and cardiac oscillations were found to have higher contributions in the short-SDS measurements than the long-SDS measurements (see Post-hoc SDS columns of Table 1). These findings have been reported by other studies, where higher coherences were observed between physiological signals collected from peripheries (blood pressure, respiratory or heart rate) and short-SDS measurements than long-SDS measurements^29,38^. In hypercapnic tasks, respiratory activity was found to have a significantly higher presence in short-SDS measurements, while myogenic activity had no difference across SDS measurements and cardiac activity having higher presence in long-SDS measurements.

Activity varied laterally across hemispheres during the cognitive task, but not during the hypercapnic task. In fact, hemisphere was not a significant predictor for the hypercapnic task. This is supported by CVR literature, which suggests that PaCO_2_ is a global stimulus that induces similar magnitude of change in CBF across all regions of PFC to a fixed input^71^. For the cognitive task, significant differences in activity were observed only in VLF band of long-SDS measurements. Firstly, no significant difference observed between hemispheres for other bands of long-SDS measurements and for all bands of short-SDS measurements is in accordance with numerous cognitive and motor studies, which have reported homogeneity of systemic activities and extracerebral fNIRS signals between right and left middle frontal areas^29,42,43,47^. Secondly, this finding verifies that the activity observed and reported in our prior publications was mainly due to task-related neuronal activity and not due to task-related extracerebral activity^100,101^. Lastly, activity being dominant in right-middle frontal areas further validates that UAS sensor operators’ tasks are highly attention demanding.

A task-moderated effect was seen within the VLF, myogenic and respiratory bands in both long- and short-SDS measurements during the cognitive task (see Fig. 5 and Supplemental Table 3). Specifically, regardless of SDS channel, VLF band activity significantly increased across tasks of the same difficulty (E1 vs E3 or H1 vs H2) but not across tasks of the same order and different difficulties (E1 vs H1 or E2 vs H2). Differences across harder tasks (H1 vs H2) had lower effect sizes than those associated with easier conditions (E1 vs E3). Such within difficulty differences in long-SDS measurements can be explained by focus, while the across difficulty can be explained by failure to meet demands posed by the more difficult task condition^102^. Specifically, if the task is too taxing, then the likelihood of the subject to make errors and more specifically disengage with the task is high^103^. Based on this, we can speculate that some subjects stopped engaging with the harder task conditions, therefore resulting in an overall lower average change across participants. Preliminary evidence of this disengagement has been shown in a previous study utilizing the same cognitive task, where behavioral outputs became worse with the harder task and were positively correlated with decreased hemodynamic changes^104^. Alternatively, similar results from short-SDS measurements may be attributed to stress relief^28,29,105,106^. These effect sizes of differences between conditions were greater in magnitude in short-SDS measurements than long-SDS measurements, suggesting a large task-evoked systemic contribution. Additionally, more task condition pairs were found to be significant in short SDS measurements, indicating the need to remove not just global scalp activity, but also task-specific systemic activity before deriving subject-disengagement related interpretations. Similar task-evoked activity as seen in the VLF band, was seen in the myogenic and respiratory bands. One interpretation of the results is that increased neuronal activity may have collectively elicited myogenic responses and the intrinsic load elicited by lack of a break given between conditions of the same difficulty may have led to over breathing, elevated consumption of oxygen and release of CO_2_. Interestingly, a significant decrease was observed from E3 to H1 in both long and short-SDS measurements across all bands. This decrease in activity may be explained by the presence of a fifteen-minute break between the two difficulty conditions. The presence of the break may have allowed for re-establishment of baseline/resting conditions prior to beginning of hard task block.

In comparison to HbO and HbT, HbR had the largest magnitude within the VLF band for both tasks. HbR has been demonstrated to be a more reliable biomarker for neuronal activation than HbO ^28,107^. This statement is further supported by the fact that during cognitive tasks HbR has higher correlation with BOLD-fMRI than HbO or HbT^108^. Higher correlations between HbR and BOLD-fMRI during CO_2_ tasks have also been reported^71,99^. However, due to its low dynamic range, HbR is more prone to motion artifacts and therefore results in an underestimation of hemodynamic activity associated with NVC or CVR^30^. The data utilized in this study had relatively high signal to noise ratios, therefore we predict that the high magnitude of peaks associated with HbR are reflective of neuronal activity and not noise. HbO and HbT indicated stronger presence of systemic factors than HbR. This is because oscillations in cardiac output induce changes in blood volume of the arteries, but not in the veins^34^. Thus, the HbR component of the fNIRS signal is generally less affected by physiological activities other than the cerebral function^28,64,107^. However, HbR is not devoid of systemic effects (see Figs. 4 and 6). Therefore, HbR should not be left uncorrected^63^. Systemic activity related to SBF was present in all biomarkers. However, unlike many studies, which have reported that SBF effects are minimal in HbR^28,41,106^, we did not observe such trend. In fact, we observed smaller differences between short and long-SDS channels, suggesting a larger extracerebral contribution to HbR signal. This finding is relatively new but has been supported by recent papers^63,109^. However, application of short SDS did not improve extraction of NVC related HbR signals^41,44,106^. Studies have reported that this may be due to a contribution that is not related to SBF^41,63^. Therefore, further investigation regarding systemic confounders on activity within the VLF band of HbR needs to be done.

Despite the promising methodology, this study results are subject to a few limitations. Due to the short duration of the hypercapnia experiments, wavelet coefficients below 0.02 Hz could not be extracted with high accuracy. Therefore, findings within VLF bands need to be further investigated with a longer protocol. The short protocol length also prevented evaluation of effects of hypercapnic vs normocapnic conditions on physiological causes, since splitting would lead to a reduction in data length to 3 min, which would prevent extraction of wavelet coefficients below 0.06 Hz. As previously mentioned a protocol of at least 15 min is needed to extract wavelet coefficients as low as 0.009 Hz^81^. Furthermore, only data from five participants were analyzed for the hypercapnic task, therefore the findings reported here are preliminary in nature. SBF and myogenic activity have been reported to be heterogeneous within extracerebral layers of frontal areas^29,42,47^. Specifically, medial frontal regions consist of scalp draining veins which lead to increased SBF effects than lateral regions. The instrumentation that was used in our study did not allow for investigation of lateral extracerebral regions. The presented CO_2_ and cognitive studies were employed in two different groups of subjects; therefore, we are not able to relate individual contributions of PaCO_2_ and VLF-induced hemodynamic changes. Hence a future study investigating both NVC and CVR within the same subject population needs to be performed. We did not collect data during the resting period, which is needed if we are to quantify non-evoked versus evoked activity within each band. We realize that the contributions observed may be a result of the long-term correlated 1/f fluctuations and therefore the physiological origin of the energy density at a certain band may be more complicated than the generally accepted and implemented separate bands in fNIRS applications as in this study^110^. To further refine the understanding of the effect of the physiological causes on fNIRS signals, in the future we plan on subtracting the spectral component of 1/f fluctuations from the frequency spectra and re-assessing with the proposed methodology. We realize that the long-SDS measurements are a combination of extracerebral and cerebral activity. Therefore, to truly assess contributions arising from each tissue layer requires separation of signals from cerebral and extracerebral layers by implementing one of the conventionally used short SDS regression methods on long-SDS measurements which we plan to perform as a future work. Lastly, to further investigate task-evoked effects, verify frequency bands in different systemic bands and identify subject’s state (in terms of stress, perceived workload, etc.), we plan to conduct similar analysis between activated and non-activated channels, coherence analysis between fNIRS and other sensors data (e.g., electrocardiography, laser doppler flowmetry) and correlational analysis between fNIRS and survey results (e.g., state trait anxiety, NASA-TLX).

In conclusion, this study indicated that spectral (physiological causes), spatial (axially—tissue layers and laterally—regions of middle frontal areas in left and right hemispheres), and temporal (task conditions) characteristics of fNIRS signals vary with the type of stimuli administered. Furthermore, we utilized continuous wavelet transform and employed multiple long- and short-source SDS channels that allows for identification of the source in signal variability, in turn, enables determination of the appropriate signal processing techniques needed to extract the signal of interest. Lastly, this study also indicates that task-evoked systemic and extracerebral effects may serve as supplementary signals of interest regarding the state of the participant while engaging with a real-world task.

## Supplementary Information

Supplementary Information.

## Data Availability

The data that supports the findings of this study are available from the corresponding authors, P.R and K.I, upon request.

## References

[1] Miller, S., Richmond, I., Borgos, J. & Mitra, K. NIRS-based noninvasive cerebrovascular regulation assessment. in *Clinical and Translational Neurophotonics; Neural Imaging and Sensing; and Optogenetics and Optical Manipulation* (eds. Madsen, S. J. et al.) vol. 9690 96900W (SPIE, 2016).

[2] Obrig H, Steinbrink J (2011). Non-invasive optical imaging of stroke. Philos. Trans. R. Soc. Math. Phys. Eng. Sci..

[3] Rostrup E, Law I, Pott F, Ide K, Knudsen GM (2002). Cerebral hemodynamics measured with simultaneous PET and near-infrared spectroscopy in humans. Brain Res..

[4] Izzetoglu M, Bunce SC, Izzetoglu K, Onaral B, Pourrezaei K (2007). Functional brain imaging using near-infrared technology. IEEE Eng. Med. Biol. Mag..

[5] Davies DJ (2015). Near-infrared spectroscopy in the monitoring of adult traumatic brain injury: A review. J. Neurotrauma.

[6] Hernandez-Meza G, Izzetoglu M, Osbakken M, Green M, Izzetoglu K (2015). Near-infrared spectroscopy for the evaluation of anesthetic depth. BioMed Res. Int..

[7] Wolf M (2002). Different time evolution of oxyhemoglobin and deoxyhemoglobin concentration changes in the visual and motor cortices during functional stimulation: A near-infrared spectroscopy study. Neuroimage.

[8] Smielewski P, Kirkpatrick P, Minhas P, Pickard JD, Czosnyka M (1995). Can cerebrovascular reactivity be measured with near-infrared spectroscopy?. Stroke.

[9] Tachtsidis, I. & Scholkmann, F. False positives and false negatives in functional near-infrared spectroscopy: issues, challenges, and the way forward. *Neurophotonics* **3** (2016). 10.1117/1.NPh.3.3.031405 PMC4791590 27054143

[10] Boas DA, Dale AM, Franceschini MA (2004). Diffuse optical imaging of brain activation: approaches to optimizing image sensitivity, resolution, and accuracy. Neuroimage.

[11] Phillips AA, Chan FH, Zheng MMZ, Krassioukov AV, Ainslie PN (2015). Neurovascular coupling in humans: Physiology, methodological advances and clinical implications. J. Cereb. Blood Flow Metab..

[12] Fantini S, Sassaroli A, Tgavalekos KT, Kornbluth J (2016). Cerebral blood flow and autoregulation: current measurement techniques and prospects for noninvasive optical methods. Neurophotonics.

[13] Liu P, De Vis JB, Lu H (2019). Cerebrovascular reactivity (CVR) MRI with CO2 challenge: A technical review. Neuroimage.

[14] Diamond GS (2006). Dynamic physiological modeling for functional diffuse optical tomography. Neuroimage.

[15] Ferrari M, Quaresima V (2012). A brief review on the history of human functional near-infrared spectroscopy (fNIRS) development and fields of application. Neuroimage.

[16] Scholkmann F, Wolf M (2013). General equation for the differential pathlength factor of the frontal human head depending on wavelength and age. J. Biomed. Opt..

[17] Tak S, Ye JC (2014). Statistical analysis of fNIRS data: A comprehensive review. Neuroimage.

[18] Huppert TJ (2016). Commentary on the statistical properties of noise and its implication on general linear models in functional near-infrared spectroscopy. Neurophotonics.

[19] Katura T, Tanaka N, Obata A, Sato H, Maki A (2006). Quantitative evaluation of interrelations between spontaneous low-frequency oscillations in cerebral hemodynamics and systemic cardiovascular dynamics. Neuroimage.

[20] Tong Y, Frederick B (2010). Time lag dependent multimodal processing of concurrent fMRI and near-infrared spectroscopy (NIRS) data suggests a global circulatory origin for low-frequency oscillation signals in human brain. Neuroimage.

[21] Sassaroli A, Pierro M, Bergethon PR, Fantini S (2012). Low-frequency spontaneous oscillations of cerebral hemodynamics investigated with near-infrared spectroscopy: A review. IEEE J. Sel. Top. Quantum Electron..

[22] Obrig H (2000). Spontaneous low frequency oscillations of cerebral hemodynamics and metabolism in human adults. Neuroimage.

[23] Holper L, Scholkmann F, Seifritz E (2015). Time-frequency dynamics of the sum of intra-and extracerebral hemodynamic functional connectivity during resting-state and respiratory challenges assessed by multimodal functional near-infrared spectroscopy. Neuroimage.

[24] Scholkmann F, Gerber U, Wolf M, Wolf U (2013). End-tidal CO2: An important parameter for a correct interpretation in functional brain studies using speech tasks. Neuroimage.

[25] Pinti P, Scholkmann F, Hamilton A, Burgess P, Tachtsidis I (2019). Current Status and issues regarding pre-processing of fNIRS neuroimaging data: An investigation of diverse signal filtering methods within a general linear model framework. Front. Hum. Neurosci..

[26] Khan RA (2020). Cortical Tasks-Based Optimal Filter Selection: An fNIRS Study. J. Healthc. Eng..

[27] Tachtsidis, I., Koh, P. H., Stubbs, C. & Elwell, C. E. Functional optical topography analysis using statistical parametric mapping (SPM) methodology with and without physiological confounds. in *Advances in Experimental Medicine and Biology.* vol. 662 237–243 (Springer, 2010). 10.1007/978-1-4419-1241-1_34 PMC4038021 20204798

[28] Kirilina E (2012). The physiological origin of task-evoked systemic artefacts in functional near infrared spectroscopy. Neuroimage.

[29] Kirilina E (2013). Identifying and quantifying main components of physiological noise in functional near infrared spectroscopy on the prefrontal cortex. Front. Hum. Neurosci..

[30] Sutoko S (2019). Denoising of neuronal signal from mixed systemic low-frequency oscillation using peripheral measurement as noise regressor in near-infrared imaging. Neurophotonics.

[31] Bauernfeind, G., Wriessnegger, S. C., Daly, I. & Müller-Putz, G. R. Separating heart and brain: On the reduction of physiological noise from multichannel functional near-infrared spectroscopy (fNIRS) signals. *J. Neural Eng.* **11**, (2014). 10.1088/1741-2560/11/5/056010 25111822

[32] Izzetoglu M, Holtzer R (2020). Effects of processing methods on fNIRS signals assessed during active walking tasks in older adults. IEEE Trans. Neural Syst. Rehabil. Eng..

[33] Wang, L., Ayaz, H. & Izzetoglu, M. Investigation of the source‐detector separation in near infrared spectroscopy for healthy and clinical applications. *J. Biophotonics* **12**, (2019). 10.1002/jbio.201900175 31291506

[34] Franceschini MA, Joseph DK, Huppert TJ, Diamond SG, Boas DA (2006). Diffuse optical imaging of the whole head. J. Biomed. Opt..

[35] Kohno S (2007). Removal of the skin blood flow artifact in functional near-infrared spectroscopic imaging data through independent component analysis. J. Biomed. Opt..

[36] Virtanen J, Noponen T, Meriläinen P (2009). Comparison of principal and independent component analysis in removing extracerebral interference from near-infrared spectroscopy signals. J. Biomed. Opt..

[37] Saager R, Telleri N, Berger A (2011). Two-detector corrected near infrared spectroscopy (C-NIRS) detects hemodynamic activation responses more robustly than single-detector NIRS. Neuroimage.

[38] Tian F (2011). Enhanced functional brain imaging by using adaptive filtering and a depth compensation algorithm in diffuse optical tomography. IEEE Trans. Med. Imaging.

[39] Zhang Y (2018). Evoked hemodynamic response estimation to auditory stimulus using recursive least squares adaptive filtering with multidistance measurement of near-infrared spectroscopy. J. Healthc. Eng..

[40] Funane T (2014). Quantitative evaluation of deep and shallow tissue layers’ contribution to fNIRS signal using multi-distance optodes and independent component analysis. Neuroimage.

[41] Sato T (2016). Reduction of global interference of scalp-hemodynamics in functional near-infrared spectroscopy using short distance probes. Neuroimage.

[42] Gagnon L (2012). Short separation channel location impacts the performance of short channel regression in NIRS. Neuroimage.

[43] Zhang Y (2015). Multiregional functional near-infrared spectroscopy reveals globally symmetrical and frequency-specific patterns of superficial interference. Biomed. Opt. Express.

[44] Gagnon L (2011). Improved recovery of the hemodynamic response in diffuse optical imaging using short optode separations and state-space modeling. Neuroimage.

[45] Dong S, Jeong J (2019). Improvement in recovery of hemodynamic responses by extended kalman filter with non-linear state-space model and short separation measurement. IEEE Trans. Biomed. Eng..

[46] Gagnon L, Yücel MA, Boas DA, Cooper RJ (2014). Further improvement in reducing superficial contamination in NIRS using double short separation measurements. Neuroimage.

[47] Erdoğan SB, Yücel MA, Akın A (2014). Analysis of task-evoked systemic interference in fNIRS measurements: Insights from fMRI. Neuroimage.

[48] Pinti P, Cardone D, Merla A (2015). Simultaneous fNIRS and thermal infrared imaging during cognitive task reveal autonomic correlates of prefrontal cortex activity. Nat. Publ. Gr..

[49] Zimeo Morais GA (2018). Non-neuronal evoked and spontaneous hemodynamic changes in the anterior temporal region of the human head may lead to misinterpretations of functional near-infrared spectroscopy signals. Neurophotonics.

[50] Scholkmann F (2014). A review on continuous wave functional near-infrared spectroscopy and imaging instrumentation and methodology. Neuroimage.

[51] Caldwell M (2016). Modelling confounding effects from extracerebral contamination and systemic factors on functional near-infrared spectroscopy. Neuroimage.

[52] Tachtsidis, I., Leung, T. S., Devoto, L., Delpy, D. T. & Elwell, C. E. Measurement of frontal lobe functional activation and related systemic effects: A near-infrared spectroscopy investigation. in *Advances in Experimental Medicine and Biology.* vol. 614 397–403 (Springer, 2008). 10.1007/978-0-387-74911-2_44 18290351

[53] Minati L, Kress IU, Visani E, Medford N, Critchley HD (2011). Intra- and extra-cranial effects of transient blood pressure changes on brain near-infrared spectroscopy (NIRS) measurements. J. Neurosci. Methods.

[54] Scholkmann, F., Wolf, M. & Wolf, U. The effect of inner speech on arterial CO2 and cerebral hemodynamics and oxygenation: a functional NIRS study. in *Oxygen Transport to Tissue XXXV* 81–87 (Springer, 2013). 10.1007/978-1-4614-7411-1_12. 10.1007/978-1-4614-7411-1_12 23852480

[55] Haeussinger FB (2014). Reconstructing functional near-infrared spectroscopy (fNIRS) signals impaired by extra-cranial confounds: An easy-to-use filter method. Neuroimage.

[56] Ranchet M, Morgan JC, Akinwuntan AE, Devos H (2017). Cognitive workload across the spectrum of cognitive impairments: A systematic review of physiological measures. Neurosci. Biobehav. Rev..

[57] Herold F, Wiegel P, Scholkmann F, Müller N (2018). Applications of functional near-infrared spectroscopy (fNIRS) neuroimaging in exercise-cognition science: A systematic, methodology-focused review. J. Clin. Med..

[58] Hakimi N, Jodeiri A, Mirbagheri M, Kamaledin Setarehdan S (2020). Proposing a convolutional neural network for stress assessment by means of derived heart rate from functional near infrared spectroscopy. Comput. Biol. Med..

[59] Holper L (2014). Physiological effects of mechanical pain stimulation at the lower back measured by functional near-infrared spectroscopy and capnography. J. Integr. Neurosci..

[60] Stefanovska A, Bracic M, Kvernmo H (1999). Wavelet analysis of oscillations in the peripheral blood circulation measured by laser Doppler technique. IEEE Trans. Biomed. Eng..

[61] Molavi B, Dumont GA (2012). Wavelet-based motion artifact removal for functional near-infrared spectroscopy. Physiol. Meas..

[62] Jang KE (2009). Wavelet minimum description length detrending for near-infrared spectroscopy. J. Biomed. Opt..

[63] Klein F, Kranczioch C (2019). Signal processing in fNIRS: A case for the removal of systemic activity for single trial data. Front. Hum. Neurosci..

[64] Lina J, Dehaes M, Matteau-Pelletier C, Lesage F (2008). Complex wavelets applied to diffuse optical spectroscopy for brain activity detection. Opt. Express.

[65] Zhang X (2015). Activation detection in functional near-infrared spectroscopy by wavelet coherence. J. Biomed. Opt..

[66] Duan L (2018). Wavelet-based method for removing global physiological noise in functional near-infrared spectroscopy. Biomed. Opt. Express.

[67] Li Z (2010). Wavelet analysis of cerebral oxygenation signal measured by near infrared spectroscopy in subjects with cerebral infarction. Microvasc. Res..

[68] Li Z (2012). Spectral analysis of near-infrared spectroscopy signals measured from prefrontal lobe in subjects at risk for stroke. Med. Phys..

[69] Li Z (2013). Age-related changes in spontaneous oscillations assessed by wavelet transform of cerebral oxygenation and arterial blood pressure signals. J. Cereb. Blood Flow Metab..

[70] Reddy, P., Richards, D. & Izzetoglu, K. Evaluation of UAS Operator Training During Search and Surveillance Tasks. In *20th International Symposium on Aviation Psychology* (2019).

[71] Amyot F (2020). Assessment of cerebrovascular dysfunction after traumatic brain injury with fMRI and fNIRS. NeuroImage Clin..

[72] Izzetoglu, K. & Richards, D. Human Performance Assessment: Evaluation of Wearable Sensors for Monitoring Brain Activity. in *Improving Aviation Performance through Applying Engineering Psychology* 163–180 (CRC Press, 2020). 10.4324/9780429492181-8.

[73] Oldfield RC (1971). The assessment and analysis of handedness: The Edinburgh inventory. Neuropsychologia.

[74] Hanzhang, L. & Peiying, L. Systems and methods for gas mixture delivery to humans inside an MRI scanner. 1–31 (2015) https://patentimages.storage.googleapis.com/b9/ae/4c/7717fd03867e6e/WO2015126795A1.pdf.

[75] Uludag K, Kohl M, Steinbrink J, Obrig H, Villringer A (2002). Cross talk in the Lambert-Beer calculation for near-infrared wavelengths estimated by Monte Carlo simulations. J. Biomed. Opt..

[76] Ayaz H (2011). Using MazeSuite and functional near infrared spectroscopy to study learning in spatial navigation. J. Vis. Exp..

[77] Izzetoglu, M. & Izzetoglu, K. Real time artifact removal. 1–9 (2014).

[78] Delpy DT, Cope M, van der Zee P (1988). Estimation of optical path length through tissue from direct time of flight measurement. Physic Med. Biol..

[79] Prahl, S. Tabulated molar extinction coefficient for hemoglobin in water. Portland: Oregon Medical Laser Center. https://omlc.org/spectra/hemoglobin/summary.html (1998).

[80] Scholkmann F, Spichtig S, Muehlemann T, Wolf M (2010). How to detect and reduce movement artifacts in near-infrared imaging using moving standard deviation and spline interpolation. Physiol. Meas..

[81] Iatsenko D, McClintock PVE, Stefanovska A (2015). Linear and synchrosqueezed time-frequency representations revisited: Overview, standards of use, resolution, reconstruction, concentration, and algorithms. Digit. Signal Process. A Rev. J..

[82] Yücel MA (2016). Mayer waves reduce the accuracy of estimated hemodynamic response functions in functional near-infrared spectroscopy. Biomed. Opt. Express.

[83] Bates D, Mächler M, Bolker BM, Walker SC (2015). Fitting linear mixed-effects models using lme4. J. Stat. Softw..

[84] Kuznetsova, A., Brockhoff, P. B. & Christensen, R. H. B. lmerTest package: Tests in linear mixed effects models. *J. Stat. Softw.* **82**, (2017).

[85] Lenth, R. emmeans: Estimated Marginal Means, aka LeastSquares Means. (2020).

[86] Friston, K. J. Statistical Parametric Mapping. in *Neuroscience Databases* 237–250 (Springer, 2003). 10.1007/978-1-4615-1079-6_16.

[87] Westfall J, Kenny DA, Judd CM (2014). Statistical power and optimal design in experiments in which samples of participants respond to samples of stimuli. J. Exp. Psychol. Gen..

[88] Tong Y, Hocke LM, Frederick BB (2019). Low frequency systemic hemodynamic “noise” in resting state BOLD fMRI: Characteristics, causes, implications, mitigation strategies, and applications. Front. Neurosci..

[89] Shiogai Y, Stefanovska A, McClintock PVE (2010). Nonlinear dynamics of cardiovascular ageing. Phys. Rep..

[90] Hosford PS, Gourine AV (2019). What is the key mediator of the neurovascular coupling response?. Neurosci. Biobehav. Rev..

[91] Xu F (2011). The influence of carbon dioxide on brain activity and metabolism in conscious humans. J. Cereb. Blood Flow Metab..

[92] Heeger DJ, Ress D (2002). What does fMRI tell us about neuronal activity?. Nat. Rev. Neurosci..

[93] Julien C (2006). The enigma of Mayer waves: Facts and models. Cardiovasc. Res..

[94] Maggio P, Salinet ASM, Robinson TG, Panerai RB (2014). Influence of CO2 on neurovascular coupling: Interaction with dynamic cerebral autoregulation and cerebrovascular reactivity. Physiol. Rep..

[95] Gauthier CJ (2013). Age dependence of hemodynamic response characteristics in human functional magnetic resonance imaging. Neurobiol. Aging.

[96] Ainslie PN, Duffin J (2009). Integration of cerebrovascular CO2 reactivity and chemoreflex control of breathing: Mechanisms of regulation, measurement, and interpretation. Am. J. Physiol. Regul. Integr. Comp. Physiol..

[97] Durduran T, Choe R, Baker WB, Yodh AG (2010). Diffuse optics for tissue monitoring and tomography. Rep. Prog. Phys..

[98] Holper L, Scholkmann F, Seifritz E (2017). Prefrontal hemodynamic after-effects caused by rebreathing may predict affective states: A multimodal functional near-infrared spectroscopy study. Brain Imag. Behav..

[99] Yang H (2020). Characterizing near-infrared spectroscopy signal under hypercapnia. J. Biophotonics.

[100] Reddy, P., Richards, D. & Izzetoglu, K. Cognitive Performance Assessment of UAS Sensor Operators via Neurophysiological Measures. In *2nd International Neuroergonomics Conference* (2018).

[101] Izzetoglu K (2014). UAV Operators Workload Assessment by Optical Brain Imaging Technology (fNIR). Unmanned Aerial Vehicles Handbook.

[102] Ayaz H (2012). Optical brain monitoring for operator training and mental workload assessment. Neuroimage.

[103] Izzetoglu K, Bunce S, Onaral B, Pourrezaei K, Chance B (2004). Functional optical brain imaging using near-infrared during cognitive tasks. Int. J. Human Computer Interact..

[104] Kerr, J., Reddy, P., Kosti, S. & Izzetoglu, K. *UAS Operator Workload Assessment During Search and Surveillance Tasks Through Simulated Fluctuations in Environmental Visibility*. *Lecture Notes in Computer Science (including subseries Lecture Notes in Artificial Intelligence and Lecture Notes in Bioinformatics)* vol. 11580 LNAI (2019).

[105] Takahashi T (2011). Influence of skin blood flow on near-infrared spectroscopy signals measured on the forehead during a verbal fluency task. Neuroimage.

[106] Yücel MA (2015). Short separation regression improves statistical significance and better localizes the hemodynamic response obtained by near-infrared spectroscopy for tasks with differing autonomic responses. Neurophotonics.

[107] Zhang X, Noah JA, Dravida S, Hirsch J (2017). Signal processing of functional NIRS data acquired during overt speaking. Neurophotonics.

[108] Cui X, Bray S, Bryant DM, Glover GH, Reiss AL (2011). A quantitative comparison of NIRS and fMRI across multiple cognitive tasks. Neuroimage.

[109] Zarei M, Ansari MA, Zare K (2019). The temporal confounding effects of extra-cerebral contamination factors on the hemodynamic signal measured by functional near-infrared spectroscopy. J. Lasers Med. Sci..

[110] Kobayashi M, Musha T (1982). 1/f fluctuation of heartbeat period. IEEE Trans. Biomed. Eng..

