# Supplementary material for: Evaluation of fNIRS signal components elicited by cognitive and hypercapnic stimuli
Source: Sci Rep. 2021 Dec 6;11:23457. doi: 10.1038/s41598-021-02076-7 (PMC8648757; doi:10.1038/s41598-021-02076-7)
Supplement: Supplementary file 1 — Supplementary Information. [file 41598_2021_2076_MOESM1_ESM.pdf]

Evaluation of fNIRS Signal Components Elicited by Cognitive and Hypercapnic Stimuli

Pratusha Reddy[1*], Meltem Izzetoglu[2,ⱶ], Patricia A. Shewokis[1,3,4,ⱶ], Michael Sangobowale[5], Ramon Diaz-Arrastia[5] and Kurtulus Izzetoglu[1,4,ⱶ]

[1] Drexel University, School of Biomedical Engineering, Science and Health Systems, Philadelphia, PA 19104 USA
[2] Villanova University, Department of Electrical and Computer Engineering, Villanova, PA 19085 USA
[3] Drexel University, Nutrition Sciences Department of College of Nursing and Health Professions, Philadelphia, PA 19104 USA
[4] Drexel University, Department of Teaching, Learning and Curriculum, School of Education, Philadelphia, PA 19104 USA
[5] University of Pennsylvania Perelman School of Medicine, Clinical TBI Research Center and Department of Neurology, Philadelphia, PA 19104 USA
[*]ylr26@drexel.edu
[ⱶ]these authors contributed equally to this work

Supplemental Material

Supplemental Table 1. Example of code used to evaluate significance effect of a term and differences across levels of a term.

Tests	Question	Explanation	Code
Log likelihood ratio	Is there a significant interaction effect of band and SDS on Ɛ	Since the data is nested, where Band is the first level and SDS is the second level, set up a model with just the first level. Therefore, build a base model using lmer () with restricted maximum likelihood (REML) set to false	M_Band = lmer(log10(Ɛ) ~ 1 + Band + (1|SubjectID),data = Dt, REML = FALSE)
		Build another model by adding the term being investigated to the base model.	M_Band_SDS = lmer(log10(Ɛ) ~ 1 + Band + Band:SDS + (1|SubjectID),data = Dt, REML = FALSE)
		Evaluate the terms contribution using ANOVA	Anova (M_Band, M_Band_SDS)
Post hoc	Are there significant differences between SDS channels per band	Build entire model with REML set to true	Model = lmer(log10(Ɛ) ~ 1 + Band + Band:SDS + Band:SDS:Hemisphere + Band:SDS:Condition + (1|SubjectID), data = Dt, REML = TRUE)
		Estimate marginal means per short and long SDS of each band using emmeans (). Set type = "response" to ensure that the outputs are back transformed to original scale	M <- emmeans (Model, specs = ~ S|Band, lmer.df = "Satterthwaite", type = "response")
		Evaluate whether SDS measurements from each band are different or not using contrast (). Set adjust to "fdr" to correct for false discovery rate.	M_c <- contrast(regrid(M), method = "pairwise", adjust = "fdr")
	How large is the effect	Calculate Cohen's d per each post hoc comparison using eff_size ()	d <- eff_size (M, sigma = sigma (Model), edf = df. residual (Model))

Supplemental Table 2. Differences in energy density values between left and right-middle frontal areas per band, source detector separation and biomarker during cognitive stimulus.

		Short-SDS				Long-SDS			
Biomarker	Band	Estimate Hemisphere		Post hoc Hemisphere		Estimate Hemisphere		Post hoc Hemisphere	
		Right β [95% CI]	Left β [95% CI]	$adj.p$	d	Right β [95% CI]	Left β [95% CI]	$adj.p$	d
ΔHbO	VLF	0.025 [0.015, 0.042]	0.023 [0.014, 0.040]	0.806	0.09	0.038 [0.022, 0.065]	0.061 [0.036, 0.103]	0.068	-0.64
	Myo	0.019 [0.011, 0.032]	0.017 [0.010, 0.028]	0.492	0.20	0.010 [0.006, 0.017]	0.010 [0.006, 0.018]	0.807	-0.07
	Resp	0.003 [0.002, 0.006]	0.002 [0.002, 0.005]	0.492	0.24	0.002 [0.001, 0.003]	0.002 [0.001, 0.003]	0.978	0.00
	Cardiac	0.010 [0.006, 0.016]	0.006 [0.004, 0.010]	0.068	0.63	0.004 [0.003, 0.007]	0.005 [0.003, 0.008]	0.492	-0.19
ΔHbR	VLF	0.005 [0.002, 0.008]	0.004 [0.002, 0.007]	0.327	0.20	0.026 [0.014, 0.048]	0.046 [0.025, 0.084]	0.142	-0.72
	Myo	0.001 [0.001, 0.002]	0.001 [0.001, 0.002]	0.274	0.25	0.001 [0.001, 0.002]	0.001 [0.001, 0.002]	0.986	0.00
	Resp	0.000 [0.000, 0.001]	0.000 [0.000, 0.001]	0.231	0.38	0.000 [0.000, 0.001]	0.000 [0.000, 0.001]	0.268	-0.28
	Cardiac	0.000 [0.000, 0.001]	0.000 [0.000, 0.001]	0.231	0.33	0.000 [0.000, 0.001]	0.000 [0.000, 0.001]	0.149	-0.53
ΔHbT	VLF	0.033 [0.019, 0.055]	0.031 [0.019, 0.052]	0.687	0.07	0.044 [0.026, 0.074]	0.055 [0.032, 0.093]	0.324	-0.31
	Myo	0.026 [0.015, 0.044]	0.023 [0.014, 0.039]	0.525	0.16	0.012 [0.007, 0.021]	0.011 [0.006, 0.018]	0.525	0.16
	Resp	0.005 [0.003, 0.008]	0.004 [0.002, 0.007]	0.525	0.21	0.002 [0.001, 0.003]	0.002 [0.001, 0.003]	0.324	0.35
	Cardiac	0.010 [0.007, 0.020]	0.008 [0.005, 0.013]	0.163	0.58	0.005 [0.029, 0.084]	0.005 [0.003, 0.009]	0.687	-0.09

β – Estimate; CI – Confidence Interval; $adj.p$ – FDR adjusted p values; l; d – Cohen's d. In post-hoc band columns, the metrics represent differences between right- and left-middle frontal areas. Negative d values mean the effect was dominant in right-middle frontal areas.

Supplemental Table 3. Differences in energy density values between conditions per band, biomarker and source detector separation measurements during cognitive stimulus. Values presented represent Cohen's d values of the post hoc difference.

Band	Biomarker	Within Difficulty						Between difficulty				Between recording	
		E1-E2		E1-E3		H1- H2		E1-H1		E2-H2		E3-H1	
		Short	Long	Short	Long	Short	Long	Short	Long	Short	Long	Short	Long
VLF	Δ HbO	-0.59	-0.47	**-0.99**	**-0.91**	**-0.89**	-0.59	0.06	0.08	-0.24	-0.04	**1.04**	**0.99**
	Δ HbR	-0.68	-0.65	**-1.36**	**-1.05**	**-0.79**	-0.32	-0.41	-0.25	-0.51	0.08	**0.95**	**0.81**
	Δ HbT	-0.50	-0.57	**-0.92**	**-0.94**	**-0.91**	-0.64	-0.01	0.12	-0.42	0.05	**0.91**	**1.06**
Myo	Δ HbO	-0.49	-0.61	-0.57	**-0.83**	-0.68	**-0.77**	0.24	0.25	0.05	0.09	**0.80**	**1.08**
	Δ HbR	-0.69	**-0.77**	**-1.18**	**-1.49**	**-0.82**	**-0.87**	-0.09	-0.21	-0.23	-0.31	**1.09**	**1.28**
	Δ HbT	-0.46	-0.59	-0.57	**-0.92**	-0.68	**-0.82**	0.19	0.25	-0.03	0.02	**0.76**	**1.17**
Resp	Δ HbO	-0.46	-0.40	-0.51	-0.57	-0.68	-0.68	0.41	0.51	0.19	0.23	**0.92**	**1.08**
	Δ HbR	-0.58	-0.47	**-0.95**	**-0.92**	-0.62	-0.61	-0.07	0.10	-0.11	-0.03	**0.88**	**1.02**
	Δ HbT	-0.44	-0.46	-0.53	**-0.80**	-0.60	**-0.71**	0.28	0.31	0.12	0.05	**0.81**	**1.11**
Cardiac	Δ HbO	0.03	0.02	0.33	0.18	-0.09	-0.18	0.56	0.48	0.45	0.28	0.23	0.30
	Δ HbR	-0.18	-0.21	-0.20	-0.20	-0.31	-0.22	0.26	0.20	0.13	0.19	0.46	0.40
	Δ HbT	0.04	0.04	0.32	0.19	-0.10	-0.13	0.55	0.43	0.41	0.26	0.22	0.24

Negative d values mean the effect was dominant in the increased with condition or session. Bolded terms represent when the post hoc comparisons was significant at an α of 0.05. Reported p values were not adjusted for false discovery rate.
